# An Amorphous Anode for Proton Battery

**DOI:** 10.1007/s40820-022-00987-2

**Published:** 2022-12-30

**Authors:** Huan Liu, Xiang Cai, Xiaojuan Zhi, Shuanlong Di, Boyin Zhai, Hongguan Li, Shulan Wang, Li Li

**Affiliations:** 1 Department of Chemistry, College of Science, Northeastern University, Shenyang, 110819 Liaoning People’s Republic of China; 2 School of Light Industry and Chemical Engineering, Dalian Polytechnic University, Dalian, 116034 Liaoning People’s Republic of China; 3 State Key Laboratory of Rolling and Automation, Northeastern University, Shenyang, 110819 Liaoning People’s Republic of China; 4 School of Metallurgy, Northeastern University, Shenyang, 110819 People’s Republic of China

**Keywords:** Proton batteries, Amorphous electrode, Multivalent cations, Remarkable cycling stability, High voltage

## Abstract

Developing advanced electrode materials is crucial for improving the electrochemical performances of proton batteries. Currently, the anodes are primarily crystalline materials which suffer from inferior cyclic stability and high electrode potential. Herein, we propose amorphous electrode materials for proton batteries by using a general ion-exchange protocol to introduce multivalent metal cations for activating the host material. Taking Al^3+^ as an example, theoretical and experimental analysis demonstrates electrostatic interaction between metal cations and lattice oxygen, which is the primary barrier for direct introduction of the multivalent cations, is effectively weakened through ion exchange between Al^3+^ and pre-intercalated K^+^. The as-prepared Al-MoO_x_ anode therefore delivered a remarkable capacity and outstanding cycling stability that outperforms most of the state-of-the-art counterparts. The assembled full cell also achieved a high voltage of 1.37 V. This work opens up new opportunities for developing high-performance electrodes of proton batteries by introducing amorphous materials.

**Supplementary Information:**

The online version contains supplementary material available at 10.1007/s40820-022-00987-2.

## Highlights

- Amorphous MoO_x_ with pre-insertion of Al^3+^ was prepared via ion-exchange protocol.
- Al-MoO_x_ anode had remarkable capacity and record-level cycling stability for proton battery.
- The proton battery using Al-MoO_x_ anode achieved a high voltage of 1.37 V.

**Supplementary Information:**

The online version contains supplementary material available at 10.1007/s40820-022-00987-2.

## Graphical abstract

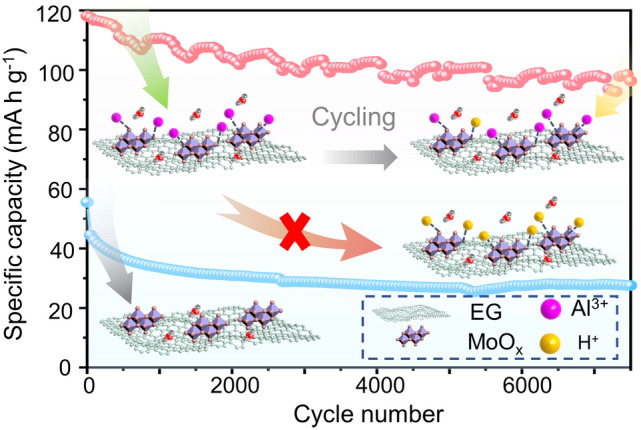

## Introduction

As one of the most important commercialized energy storage technologies, lithium-ion batteries have received impressive development for the applications in transportation vehicles, grid service systems and renewable energy sectors due to their high energy density and stable energy output [1–4]. However, the lack of lithium resources and the safety issues caused by flammable organic electrolytes inspire the search for potential alternatives [5–9]. Proton batteries (PBs) with aqueous electrolytes are attracting the increasing attentions because of their advantages for high intrinsic safety, low cost and environmental benignity [10, 11]. More importantly, the smallest ionic radius and lightest weight of charge carrier (H^+^) among all cations endow PBs with extremely fast diffusion rate and thus high rate capability based on the Grotthuss proton conduction [12–18]. Unfortunately, the primary issues for PBs are their inferior cycling performances and low average voltage, while low operating voltage also strongly restricts the elevation of energy density and selection of electrode materials. Among different optimization strategies including electrolyte engineering, current collector design, etc. [19, 20], the development of advanced anode materials is viewed as a direct and promising avenue to address these issues and promote the commercialization of PBs [15, 21]. However, considering the occurrence of hydrogen evolution reaction and the acidic electrolytes [19], it is still challenging to obtain the appropriate anode materials with high performances.

Currently, the anode materials for PBs are primarily crystalline materials, including metal oxide, metal carbide and organic materials [22–25]. Among them, molybdenum oxide (MoO_3_) is one of the most common choices due to its merits of high theoretical capacity and excellent proton conduction capability [13]. However, the low structural stability in acidic electrolytes can lead to material dissolution and thus inferior cycling performances [26]. Meanwhile, more than 80% capacity delivered by crystalline MoO_3_ is stored at the potential above 0 V, which is harmful to enhance the energy density of the full cell. The amorphous materials with high mechanical strength and strong chemical stability may provide some advantageous features for energy storage compared with their crystalline counterparts [27, 28]. For example, the short-range ordered structure of amorphous materials can facilitate Li^+^ diffusion and enhance atomic/ionic mobility within electrodes [20]. However, the desirable electrochemical characteristics are always compromised by their inherent low electrical conductivity which will significantly reduce the electrochemical performances of devices [29]. The introduction of alkali metal ions may enhance electrical conductivity of host amorphous materials [29–31], while monovalent cations with smooth intercalation kinetics cannot effectively resist the exchange of H^+^ during discharging/charging, which deteriorates the cycling performances of electrodes. Meanwhile, with the increase in valence state, the effective intercalation of metal cations is difficult to be achieved due to the strong electrostatic interactions arising from the large charge densities [32, 33]. Amorphous materials have been widely studied in electrochemical energy storage due to their unique properties. Particularly for the insertion-type electrodes, amorphous materials can provide sufficient active sites, reduce the diffusion distance of the ion, remit the volume expansion and relieve the structural stress during insertion, which are favorable for achieving efficient charge storage of electrodes [34, 35]. To the best of our knowledge, the works that focus on the development of advanced amorphous anode materials for PBs are rarely reported [36].

Herein, we propose amorphous anode materials of aqueous proton battery with ultralong cycling stability. An ion-exchange strategy was performed to introduce Al^3+^ into the amorphous MoO_x_ structure from K^+^ pre-intercalated material, which solved the issue that the substantial multivalent metal cations cannot be directly inserted into the amorphous anode. The combined theoretical calculations and experimental analysis reveal that the introduction of Al^3+^ can effectively activate MoO_x_ by allowing more H^+^ insertion/extraction for reversible energy storage to increase its capacity and inhibit the accompanied irreversible reactions to enhance its cycling performance. Accordingly, the as-prepared amorphous Al-MoO_x_ demonstrates a remarkably enhanced capacity of 159 mAh g^−1^ at 0.3 A g^−1^ as well as excellent cycling performance with a capacity retention of 81.2% even after 7,500 cycles at 12 A g^−1^, which are far better than the amorphous MoO_x_ and its crystalline counterparts. The assembled Al-MoO_x_//MnO_2_ full cell also shows a high average voltage of 1.37 V, which surpasses the values of most reported PBs, and thus delivers a high energy density of 160.2 Wh kg^−1^ at a power density of 184.3 W kg^−1^. This work proposes a prototype new anode material for PBs with all-sided high electrochemical performances.

## Experimental Section

### Materials Synthesis

#### ***Synthesis of MoO***_***x***_*** and M-MoO***_***x***_

The graphite paper was firstly exfoliated through a three-electrode configuration with graphite foil (GF, 1 × 1 cm^2^) as the working electrode, saturated calomel electrode (SCE) as the reference electrode and Pt plate as the counter electrode, respectively. The exfoliated graphite (EG) substrate was obtained after exfoliation in 100 mg mL^−1^ lithium perchlorate (99.9%, Aladdin) in propylene carbonate (PC, 99.7%, Sinopharm Chemical Reagent Co., Ltd.) solution via constant potential at − 2.4 V for 10 s. Electrochemical deposition of MoO_x_ was then prepared in a three-electrode cell via constant potential held at -1 V for 270 s with EG as the working electrode, GF and SCE as the counter and reference electrodes, respectively. The electrolyte is a mixture of aqueous solution containing 0.1 M sodium molybdate (Na_2_MoO_4_, 99%, Sinopharm Chemical Reagent Co., Ltd.) and 0.1 M ammonium acetate (CH_3_COONH_4_, 98%, Sinopharm Chemical Reagent Co., Ltd.). After that, it was converted to K-MoO_x_ by cyclic voltammetry (CV) technique at the potential range of -0.4 to -1 V with the scan rate of 5 mV s^−1^ for 500 cycles using 3 M KCl (99.5%, Sinopharm Chemical Reagent Co., Ltd.) solution as the electrolyte. Finally, Al-MoO_x_ was obtained in 2 M AlCl_3_ (98%, Sinopharm Chemical Reagent Co., Ltd.) solution with ion-exchange method by CV at the potential range of 0 to -0.7 V with the scan rate of 2 mV s^−1^ for 10 cycles using the obtained K-MoO_x_ (on EG) as the initial working electrode. The synthetic procedures of Mg-MoO_x_, Ca-MoO_x_ and Sr-MoO_x_ were similar to that of Al-MoO_x_ but only using different potential ranges and electrolytes (Mg-MoO_x_: − 0.2 to − 1.15 V in 2 M MgCl_2_; Ca-MoO_x_: − 0.2 to − 1.2 V in 2 M CaCl_2_; and Sr-MoO_x_: -0.2 to -1.1 V in 2 M SrCl_2_).

#### ***Synthesis of M-MoO***_***x***_***-L***

Al-MoO_x_-L was prepared by CV technique at the potential range of 0 to -0.7 V with the scan rate of 2 mV s^−1^ for 500 cycles in 2 M AlCl_3_ solution using MoO_x_ as the starting material. The synthetic procedures of Mg-MoO_x_-L, Ca-MoO_x_-L, Sr-MoO_x_-L were similar to that of Al-MoO_x_-L by using CV technique to insert cations to MoO_x_ directly with different potential ranges (Mg-MoO_x_-L: − 0.2 to − 1.15 V in 2 M MgCl_2_; Ca-MoO_x_-L: − 0.2 to − 1.2 V in 2 M CaCl_2_; and Sr-MoO_x_-L: − 0.2 to − 1.1 V in 2 M SrCl_2_).

#### ***Synthesis of MnO***_***2***_

Electrochemical deposition of MnO_2_ was prepared in a three-electrode cell at 5 mA cm^−2^ for 0.4 h with EG as the working electrode, GF and SCE as the counter and reference electrodes, respectively. The aqueous deposition electrolyte contains 2 M MnSO_4_ (98%, Sinopharm Chemical Reagent Co., Ltd.) with using sulfuric acid to adjust the pH to be 0.4.

### Materials Characterizations

The phase composition and crystal structure of the materials were analyzed by X-ray diffraction (XRD, PANalytical X’Pert Pro, Netherlands) using Cu/Kα as the radiation source in the 2θ range of 10°-70°. The morphology and microstructure of the materials were investigated by scanning electron microscopy (SEM, HITACHI, SU8010, Japan) equipped with an energy-dispersive X-ray spectroscopy (EDS, Carl Zeiss, Germany), and transmission electron microscopy (TEM, JEOL, JEM-ARM200F, Japan). The elemental contents of active materials were characterized by inductively coupled plasma light emission spectrometer (ICP-OES, PerkinElmer, Optima 4300DV, USA). The Raman spectra were recorded by using the Raman spectrometer (Raman, Renishaw inVia, UK) with a 514 nm laser light source. The X-ray photoelectron spectroscopy (XPS, Thermo Scientific Escalab, ESCALAB 250Xi, USA) was used to investigate the chemical states of active materials, and the corresponding spectra were calibrated using the C 1* s* peak at 284.6 eV as the reference. The electron spin resonance (EPR) measurements were performed by an EPR spectrometer (A300-10/12, Bruker, Germany) at 77 K.

### Electrochemical Measurements

All electrochemical measurements were performed at room temperature. The half-cell performances were then evaluated through a three-electrode configuration by using SCE and GF as the reference and counter electrodes, respectively. The amount of electrolyte in half-cell is 15 mL. The working electrode was directly used from the as-prepared materials that have been deposited on EG as shown above. The mass loading of MoO_x_, K-MoO_x_ and Al-MoO_x_ was obtained by calculating the mass change of EG substrate before and after electrochemical treatment. The mass loading of active materials in the electrode was controlled to be about 1.7 mg cm^−2^. A H-type full cell (length: 6 cm, width: 3 cm, and height: 5 cm) was assembled by using the as-prepared Al-MoO_x_ (3 mg cm^−2^) as the anode, MnO_2_ (pre-deposited on EG, 2 mAh cm^−2^) as the cathode with 5 mL 2 M AlCl_3_ as the electrolyte of anode and 5 mL 2 M MnSO_4_ (adjusted with the same pH value to that of 2 M AlCl_3_ by sulfuric acid) as the electrolyte of cathode. Nafion 117 proton exchange membrane was used as the separator. The CV and galvanostatic charge–discharge curves as well as rate capabilities and cycling performances were analyzed on the CHI660D electrochemical workstation (Shanghai Chenhua instrument Co., Ltd). The potentiostatic intermittent titration technique (PITT) was performed in 2 M AlCl_3_ from open circuit potential (OCP) to -0.6 V with a potential step of 10 mV for 15 min.

Specific capacity of the single electrode is calculated using Eq. 1 based on GCD profiles:

1 $$C_{{\text{S}}} = \frac{I \times t}{{m \times 3.6}}$$

where *C*_s_ is the specific capacity (mAh g^−1^), *I* is the discharge current (mA), *t* is the discharge time (s), and *m* is the mass loading of active material (mg).

Specific capacity of the full cell is obtained from Eq. 2:

2 $$C_{{\text{f}}} = \frac{I \times t}{{m \times 3.6}}$$

where *C*_f_ is the specific capacity (mAh g^−1^), and *m* is the mass loading of active materials of the anode (mg).

Energy density and power density of the full cell are calculated based on Eqs. 3 and 4, respectively:

3 $$E_{f} = \frac{C \times V}{m}$$

4 $$P_{{\text{f}}} = \frac{{3.6 \times E_{{\text{f}}} }}{t}$$

where *E*_f_ and *P*_f_ are the energy density (Wh kg^−1^) and power density (kW kg^−1^) of the full cell, respectively; C is the discharge capacity (mAh); V is the average discharge voltage (V); m is the total mass (g) of anode (Al-MoO_x_) and cathode (the consumed MnO_2_); and *t* is the discharge time (s).

### Theoretical Calculations

The formation mechanism of Al-MoO_x_ structure and the storage mechanism of H^+^ in Al-MoO_x_ were investigated by density functional theory (DFT) using the projected augmented wave (PAW) method in the Vienna Ab-Initio Simulation Package (VASP). The exchange correlation between core and valence electrons was described by generalized gradient approximation (GGA) using the spin-polarized Perdew–Burke–Ernzerhof (PBE) formulation. Besides, the dispersion interactions were also taken into account using DFT-D3 methodology with a cutoff energy of 450 eV. The Brillouin zone was sampled by the Monkhorst–Pack method with a 3 × 3 × 1 k-point grid. Partial occupancies of the Kohn–Sham orbitals were allowed using the Gaussian smearing method with a width of 0.05 eV. A geometry optimization was considered convergent when the energy convergence criterion is 1 × 10^–4^ eV and the force certification is 0.05 eV Å^−1^.

## Results and Discussion

### Structural Analysis of Al-MoO_x_

**Fig. 1:**
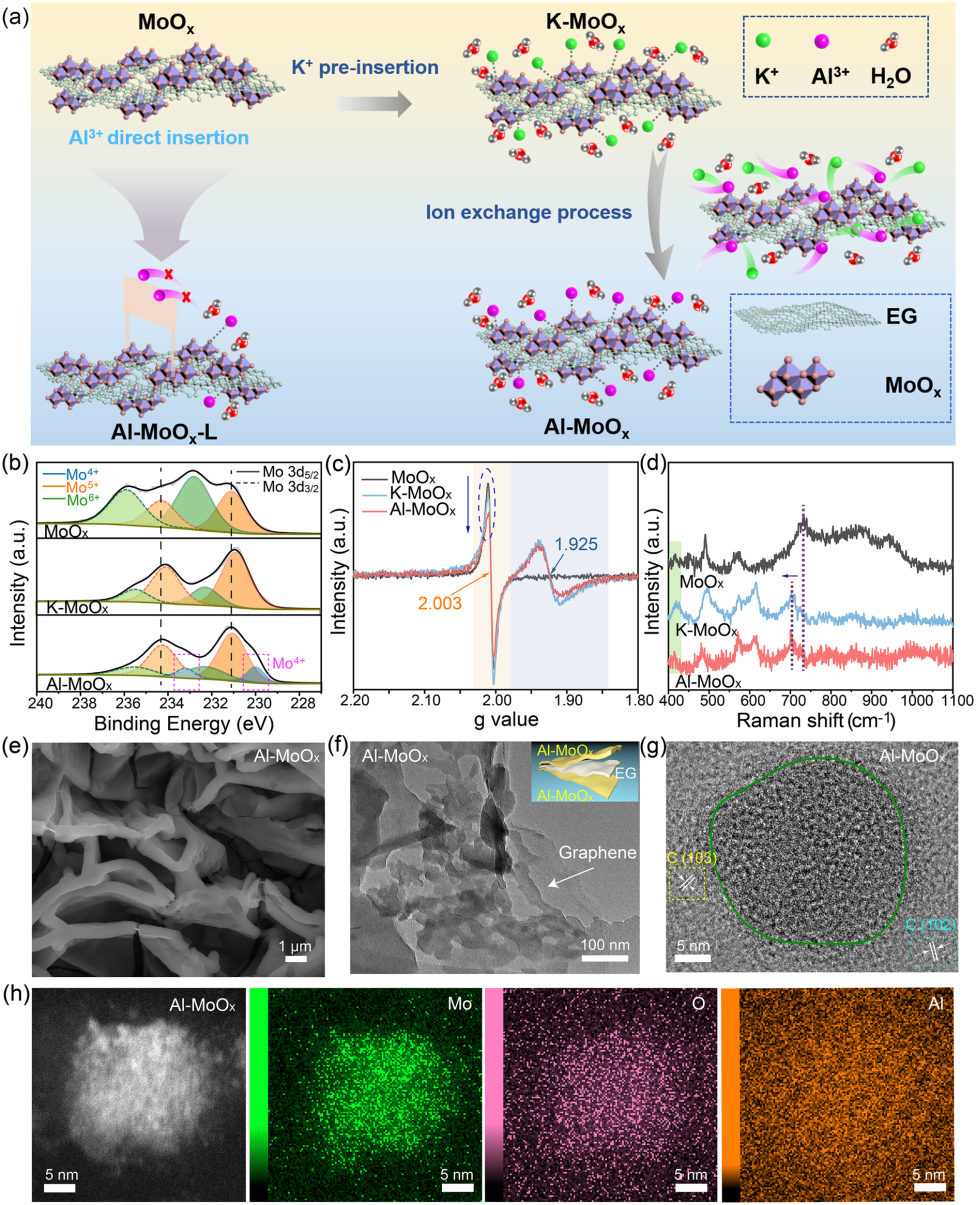
Design and structural characterization of Al-MoO_x_. **a** Schematic illustration of the design and preparation of Al-MoO_x_ from ion exchange and Al-MoO_x_-L from direct insertion. **b** Mo 3*d* XPS, **c** EPR and **d** Raman spectra of MoO_x_, K-MoO_x_ and Al-MoO_x_. **e** SEM, **f** low-resolution bright-field TEM (with the structural schematic as the inset), **g** HRTEM, **h** STEM images and the corresponding elemental mappings of Mo (green), O (pink) and Al (orange) for Al-MoO_x_

Herein, an ion-exchange strategy was conducted to introduce Al^3+^ into the amorphous molybdenum oxide (MoO_x_) (Fig. 1a). Instead of directly inserting Al^3+^ that has high kinetic barrier for entering into the lattice of MoO_x_, K^+^ ion was firstly introduced (K-MoO_x_) to partially screen the charge of lattice oxygen and reduce the electrostatic interaction of Al^3+^ with lattice oxygen through a cyclic voltammetry (CV) technique. K^+^ was confirmed to be more liable to insert into the structure of MoO_x_ than Al^3+^ and other monovalent cations (Na^+^ and Li^+^) with the detailed experimental evidences shown in the subsequent section (Fig. S1). Another CV (10 cycles for the entire substitution) was then performed for K-MoO_x_ in 2 M AlCl_3_ to allow the fully insertion of Al^3+^ along with H^+^ by exchanging K^+^ from MoO_x_ (Al-MoO_x_) (Fig. S2). Herein, K^+^ serves as a “bridging connector” by dividing the Al^3+^ insertion process into two steps and providing a medium station with lower kinetic barrier, which effectively promotes the entry of Al^3+^ into the structure of MoO_x_.

The structural changes of the host material (MoO_x_) induced by ion insertion were then traced in detail. By analyzing the Mo oxidation state from MoO_x_, K-MoO_x_ to Al-MoO_x_ (Fig. 1b) based on the Mo 3*d* X-ray photoelectron spectroscopy (XPS) spectra, it can be clearly observed that the contents of Mo^5+^ (deconvoluted into the one pair of doublets at 231.1 eV (3*d*_5/2_) and 234.3 eV (3*d*_3/2_)) and Mo^6+^ (232.7 eV (3*d*_5/2_) and 235.9 eV (3*d*_3/2_)) change significantly. Mo^5+^ content increases from 40% (MoO_x_) to 76% (K-MoO_x_) accompanied with the valence state of Mo from 5.6 to 5.24 (Table S1). Meanwhile, the shift of Mo^6+^ and Mo^5+^ peaks of K-MoO_x_ to the low binding energy indicates the formation of the interaction of K–O–Mo [28, 29]. After the fully exchange of Al^3+^ with K^+^, Mo^6+^ content did not change, while Mo^5+^ content decreased due to the irreversible insertion of H^+^ which has been widely found in crystalline MoO_3_ [13, 26]. This exchange led to the appearance of new pair of spin–orbit doublets at 230.0 and 233.2 eV that are related to Mo^4+^ (with a content of 13%). The valence state of Mo in Al-MoO_x_ was further reduced to 5.1.

In addition to the Mo state changes through the guest metal ion insertion, the evolution of oxygen vacancies (OVs) in MoO_x_ was also explored. Electron spin resonance (EPR) results (Fig. 1c) show a slight decrease in the characteristic signal of OVs at g-value of 2.003 for Al-MoO_x_, indicating that the reduction of Mo valence state is induced by Al^3+^ insertion rather than OVs introduction [37]. The Mo valence state was further experimentally determined by the thermogravimetric analysis (TGA) (Fig. S3), and a value of 5.7 was obtained for Mo in MoO_x_ (thus converted to MoO_2.85_). This result is consistent with that from XPS (5.6 shown above), while the slight difference originates from proton loss in TGA that is maintained in the real case as shown by XPS [38]. Furthermore, a new signal at g-value of 1.925 is observed in K- and Al-MoO_x_, signifying the formation of low-valance Mo species [39, 40]. The inserted metal ion was determined through the inductively coupled plasma optical emission spectroscopy (ICP-OES) and expressed as the form of molar ratio between M (M = K and Al) and Mo (M/Mo). The value of Al/Mo in Al-MoO_x_ is measured to be 0.15 which is about 1/3 of K/Mo in K-MoO_x_ (0.4) (Table S2). On the contrary, the value of Al/Mo in the direct-inserted sample (Al-MoO_x_-L) only reaches 0.05, which provides the direct evidence for the effectiveness of the ion-exchange method for introducing more Al^3+^ into the structure of MoO_x_. X-ray diffraction (XRD) patterns confirm the amorphous structure of MoO_x_, K-MoO_x_ and Al-MoO_x_ with no peaks observed except patterns from graphite carbon substrate (Fig. S4). Raman spectra (Fig. 1d) further verify the successful insertion of K^+^ and Al^3+^ with the apparent shift of Mo_3_–O peaks at 734 cm^−1^ to the low wavenumber compared with those of MoO_x_ [41, 42]. Note the weakened intensities of these peaks indicate the change of electronic structure with the insertion of metal ions and a semiconductor-to-metal transition may occur [43, 44]. The as-prepared Al-MoO_x_ shows a flake-like morphology (Fig. 1e) that is similar to MoO_x_ (Fig. S5) and K-MoO_x_ (Fig. S6) with the only difference in the wall thickness. Transmission electron microscopy (TEM) image further reveals that Al-MoO_x_ flake electrochemically grown on graphite nanosheet surface is composed of nanoparticles with sizes in the range of 20 ~ 50 nm and substantial pores can be found (Fig. 1f). In accordance with XRD result (Fig. S4), the high-resolution TEM (HRTEM) image further confirms the amorphous structure of Al-MoO_x_ without lattice fringes observed (Fig. 1g). The high-angle-annular-dark-field scanning transmission electron microscopy (HAADF-STEM) image of Al-MoO_x_ and the corresponding elemental mappings of Mo, O and Al demonstrate the homogeneous distribution of Al^3+^ in MoO_x_ (Figs. 1h and S7).

### Proof of Structural Evolution and Potential Applicability

**Fig. 2:**
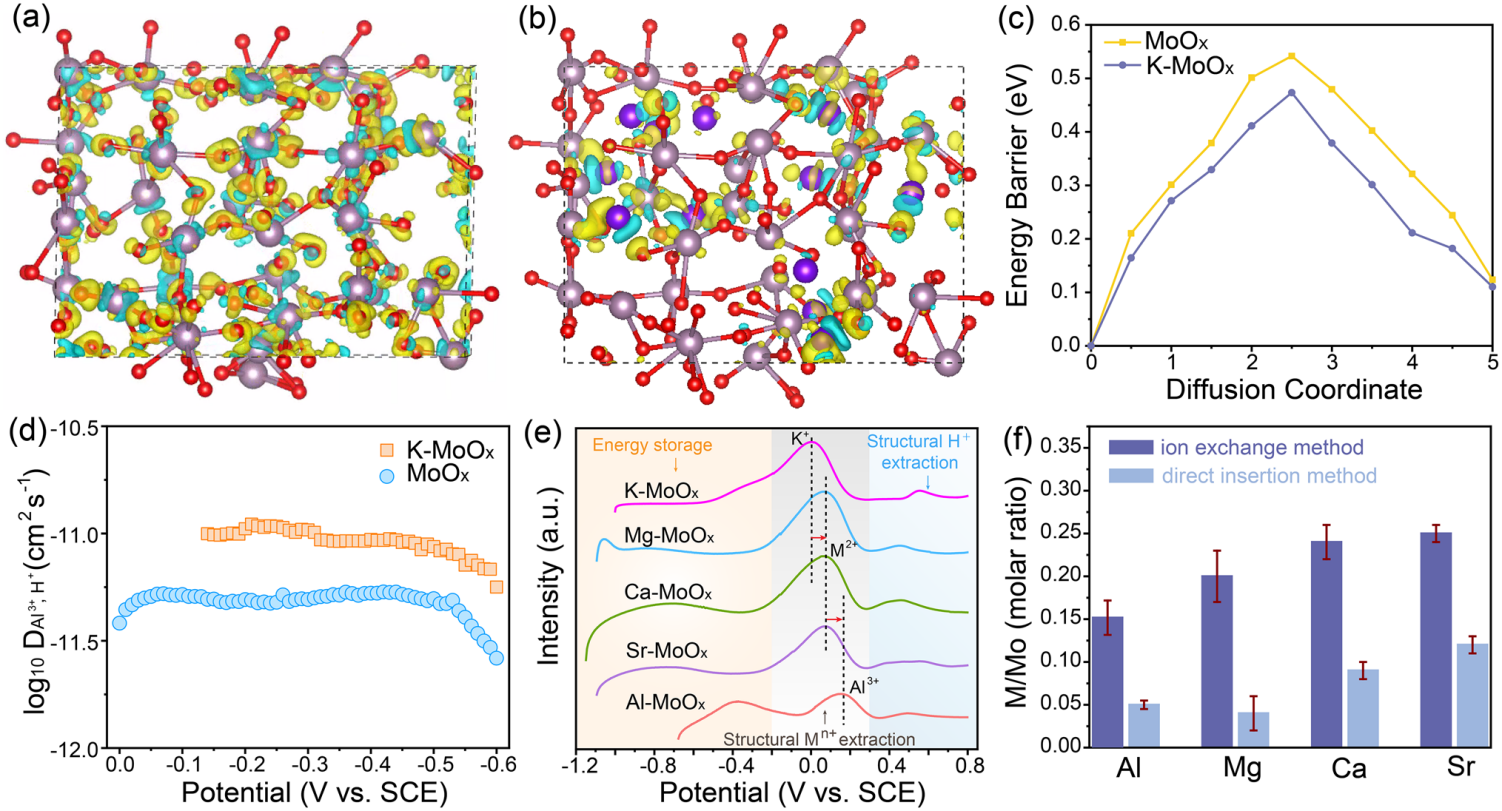
Ion-exchange mechanism and applicability. Differential charge density of **a** MoO_x_ and **b** K-MoO_x._ (The value of the isosurface is 0.005 e Å^−3^.) The yellow and blue regions refer to electronic charge accumulation and depletion. The gray, red and purple balls refer to Mo, O and K atoms, respectively. **c** Comparison of diffusion energy barrier curves for Al ion in MoO_x_ and K-MoO_x_. **d** The corresponding Al^3+^ and H.^+^ diffusion coefficients of K-MoO_x_ and MoO_x_. **e** LSV curves of structural cation extraction for M-MoO_x_ (M = Al, Mg, Ca, Sr and K). **f** ICP-OES results of M-MoO_x_ by ion-exchange and direct insertion methods (M = Al, Mg, Ca and Sr)

To provide the in-depth understanding for the fundamental ion-exchange mechanism, DFT calculations were conducted to explore the structural changes of MoO_x_ before and after metal ion insertion (Fig. S8). The differential charge density analysis for MoO_x_ and K-MoO_x_ (Fig. 2a, b) shows charge accumulation regions around oxygen are redistributed through K^+^ insertion. The corresponding Bader charge calculations reveal that the charge around O sites decreases by > 12% from − 1.65 ~ − 1.79 e (for MoO_x_) to − 1.49 ~ − 1.58 e (for K-MoO_x_) (Fig. S9), demonstrating that the presence of structural K^+^ can partially shield the charge of lattice oxygen and therefore reduce the electrostatic interaction to the guest cations. On this basis, we further calculate the diffusion energy barrier of Al^3+^ into MoO_x_ as well as K-MoO_x_ (Fig. 2c). The result clearly shows the diffusion energy of Al^3+^ is notably weakened in K-MoO_x_ compared with that in the pristine MoO_x_ with the same diffusion paths, indicating that the pre-insertion of K^+^ can lead to an easier entry of Al^3+^ into the structure of MoO_x_. This result also explains the reason that the amount of Al^3+^ in Al-MoO_x_ is more than 3 times of that in the direct insertion counterpart (Al-MoO_x_-L) as shown in Table S2. The possible diffusion paths of Al^3+^ in MoO_x_ and K-MoO_x_ are also listed in Fig. S10. The potentiostatic intermittent titration technique (PITT) was performed to investigate the kinetic behaviors of these two structures (Fig. S11), and the corresponding chemical diffusion coefficient *D* values of cations in K-MoO_x_ at each potential are about two times higher than that in MoO_x_ (Fig. 2d), confirming the faster Al^3+^ diffusion process in K-MoO_x_ that is consistent with the theoretical calculations.

We further studied exchange mechanism of Al^3+^ to K^+^ upon CV oxidized stage and investigated the potentially broad applicability of this method for other cations such as Mg^2+^, Ca^2+^ and Sr^2+^. Linear sweep voltammetry (LSV) curves of M-MoO_x_ (M = K, Mg, Ca, Sr, Al) were recorded to analyze the electrochemically structural stability of different materials and determine their possibility for ion-exchange (Fig. 2e). It can be expected that if the cations have higher structural stability, the higher potential should be applied to completely extract them out from host materials. The LSV curves can be divided into three regions with < -0.2 V that is electrochemical energy storage process of molybdenum oxide for reversible insertion/extraction of M^n+^, − 0.2 ~ 0.3 V for the irreversible extraction of structural cations (M^n+^), and > 0.3 V for the irreversible extraction of structural protons (at 0.5 V). (The details can be found for Figs. S12 and S13 and the supplementary texts.) The oxidation peak for extracting structural K^+^ in K-MoO_x_ is located at 0 V and then shifted to the higher potential for Mg^2+^, Ca^2+^, Sr^2+^ and Al^3+^. This indicates multivalent metal cations with higher structural stability tend to be remained in MoO_x_, confirming their exchange capabilities with K^+^. The H^+^ ions mainly participate in energy storage process rather than ion exchange within first several cycles of CV. After several CV cycles, the ion-exchange mechanism is summarized as follows:

5 $${\text{K}}_{{{0}{\text{.4}}}} {\text{MoO}}_{{{2}{\text{.85}}}} {\; +\; 0}{\text{.4/nM}}^{{\text{n + }}} \to {\text{M}}_{{{0}{\text{.4/n}}}} {\text{MoO}}_{{{2}{\text{.85}}}} { \;+\; 0}{\text{.4K}}^{ + }$$

The comparison of metal concentration from direct insertion and ion-exchange method is listed in Fig. 2f. All metal ions show remarkably increased insertion amount compared with their counterparts from direct insertion, enabling the effective inhibition of hydrogen evolution reaction and thus significantly elevating the capacity for energy storage (Fig. S14). The subsequent XPS analysis also revealed the appearance of Mo^4+^ with the insertion of Mg^2+^, Ca^2+^ and Sr^2+^ (Fig. S15 and Table S3), which is similar to the case of Al^3+^ shown in Fig. 1b.

### Electrochemical Performances of Half-cell Assemblies and Charge Storage Mechanism

**Fig. 3:**
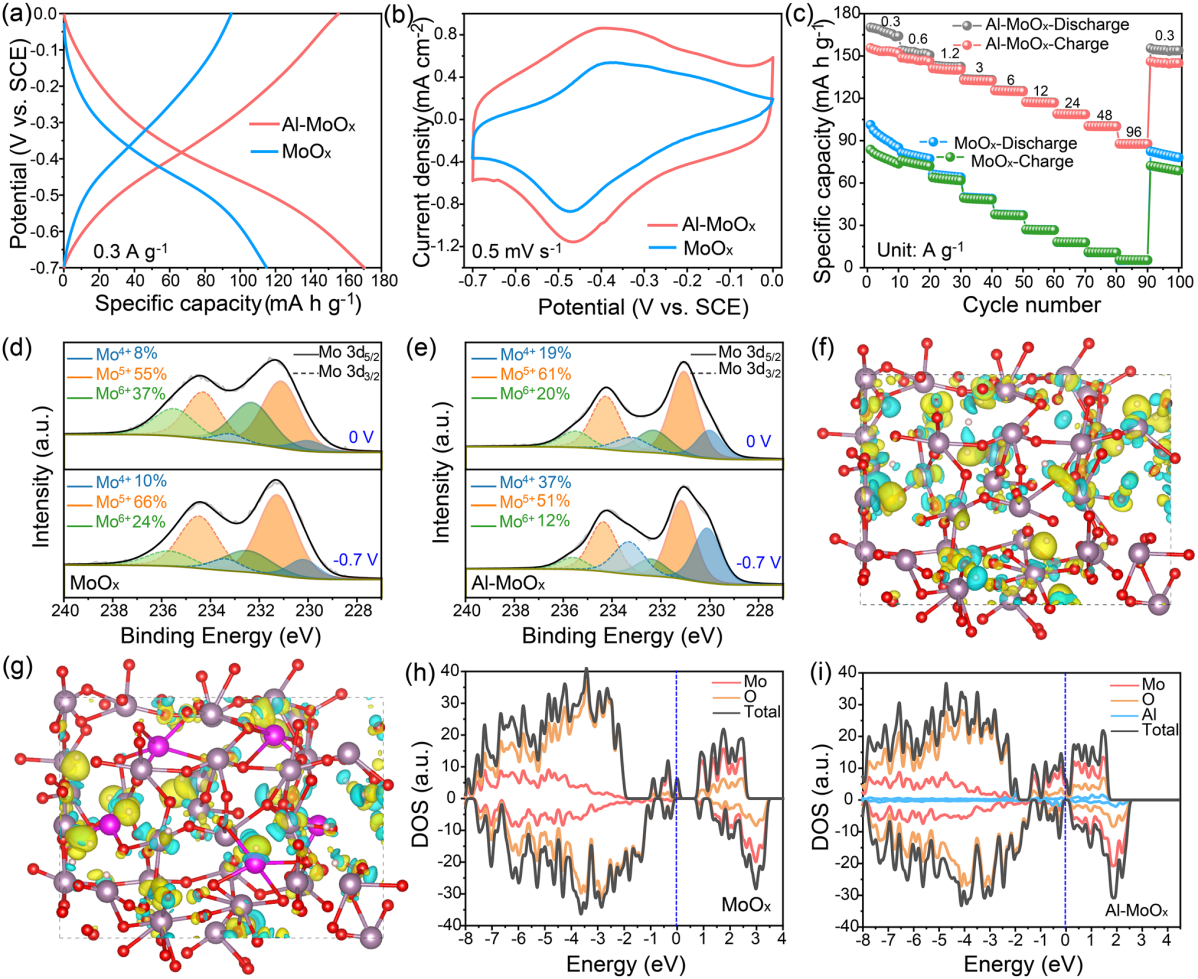
Hydrogen-ion storage performances and mechanism. **a** GCD curves at 0.3 A g^−1^, **b** CV curves at 0.5 mV s^−1^ and **c** rate capability of MoO_x_ and Al-MoO_x_. Mo 3*d* XPS spectra of different charge states of **d** MoO_x_ and **e** Al-MoO_x_. Differential charge density of hydrogen-ion insertion in **f** MoO_x_ and **g** Al-MoO_x_. The yellow and cyan regions refer to electronic charge accumulation and depletion. The gray, red, purple, pink and white balls refer to Mo, O, K, Al and H atoms, respectively. Density of states for **h** MoO_x_ and **i** Al-MoO_x_

The electrochemical performances of MoO_x_ and Al-MoO_x_ were investigated in 2 M AlCl_3_ aqueous electrolyte at a pH of 0.4. Herein, the strong acidic environment with high concentration of hydrogen ions that allows the sufficient charge carriers for electrochemical energy storage arises from the hydrolysis of partial Al^3+^, while Al^3+^ in electrolyte can effectively suppress the decomposition of Al-MoO_x_ to MoO_x_. Both MoO_x_ and Al-MoO_x_ presented nearly the same hydrogen storage behaviors with highly similar shape of galvanostatic charge–discharge (GCD) and CV curves, except for the different capacities and covered regions (Fig. 3a, b). Al-MoO_x_ delivered a specific capacity of 159 mAh g^−1^ at a current density of 0.3 A g^−1^, much higher than that of MoO_x_ (85 mAh g^−1^). A pair of redox peaks at -0.4 V that is related to insertion/extraction of proton can be found in both CV curves. The electrochemical performance elevation of Al-MoO_x_ can be clearly observed for its rate capabilities (Figs. 3c and S16), and a specific capacity of 88 mAh g^−1^ is delivered by Al-MoO_x_ even at 96 A g^−1^, corresponding to 55% capacity retention with 320 times increase of current density. As a comparison, only 5.5 mAh g^−1^ capacity is remained for MoO_x_ at 96 A g^−1^ (Fig. S17), indicating that MoO_x_ itself is almost inactive for the ultrafast charge storage. Meanwhile, though showing inferior reactivity to Al-MoO_x_, K-MoO_x_ still presented some electrochemical response of 157 mAh g^−1^ at 0.3 A g^−1^ (Fig. S18), confirming that the inserted structural cations can improve the hydrogen storage capability of amorphous molybdenum oxide.

Ex situ techniques were used to investigate the charge storage mechanism of Al-MoO_x_. The corresponding XRD patterns (Fig. S19) show no crystalline-phase reactions occur during discharge/charge. The previous work reports the primary ion storage mechanism in crystalline electrode is the intercalation of Al^3+^ into the host materials rather than hydrogen-ion storage [45]. Herein, we measured the content change of Al/Mo molar ratio at discharge state (Fig. S20) and the result shows the amount of reversible Al^3+^ is small (increased by 0.05), indicating that ~ 85% stored charge is dominated by insertion/extraction of proton in the amorphous electrode shown herein. Meanwhile, the CV curves of Al-MoO_x_ in the same concentration of electrolytes (0.5 M AlCl_3_) but with different adjusted pH values (0.5, 1, and 2.3) show its redox peak gradually shifts toward low potential (from − 0.4 to − 0.55 V) with the increased pH values (Fig. S21a), furthering confirming that the primary charge storage mechanism is H^+^-involved reactions in which electrode potential follows Nernst equation [46]. The slight Al^3+^ storage in this case is considered to be in the form of pseudocapacitance since the redox peak intensity increases with the reduction of Al^3+^ concentration in the electrolytes with the same pH values (Fig. S21b). Ex situ XPS analysis for MoO_x_ at charge (0 V, Fig. S19e dot) and discharge state (− 0.7 V, Fig. S19c dot) reveals the content of Mo^6+^, Mo^5+^ and Mo^4+^ changes from 37%, 55% and 8% at 0 V to 24%, 66% and 10% at − 0.7 V, respectively (Fig. 3d and Table S4), demonstrating that the primary charge storage of MoO_x_ is dominated by Mo^5+^/Mo^6+^ redox couple (since the change of Mo^4+^ content is negligible). Note herein the presence of low content Mo^4+^ in MoO_x_ arises from the irreversible proton introduction during discharge/charge rather than the inherent state, and it is not electrochemically active [29, 47]. In contrast, the content of Mo^6+^, Mo^5+^ and Mo^4+^ for Al-MoO_x_ changes from 20%, 61% and 19% at 0 V to 12%, 51% and 37% at − 0.7 V (Fig. 3e), which indicates the charge storage of Al-MoO_x_ also originates from Mo^4+^/Mo^5+^ that is activated through the insertion of Al^3+^ (Fig. S22). Based on the above analysis, the charge storage mechanism of Al-MoO_x_ can be expressed as follows:

6 $${\text{Al}}_{{{0}{\text{.15}}}} {\text{MoO}}_{{{2}{\text{.85}}}} { + 0}{\text{.75H}}^{ + } + {0}{\text{.05Al}}^{{3 + }} { + 0}{\text{.9e}}^{ - } \leftrightarrow {\text{H}}_{{{0}{\text{.75}}}} {\text{Al}}_{{{0}{\text{.2}}}} {\text{MoO}}_{{{2}{\text{.85}}}}$$

DFT calculations were then performed to investigate the interactions between inserted protons (H^+^) and molybdenum oxide. The differential charge density analysis shows the insertion of H^+^ changes charge distribution within the host framework (MoO_x_) (Fig. 3f, with the structure of original MoO_x_ listed in Fig. 2a). The charge transfer from MoO_x_ to H^+^ is about 0.65e for one H^+^ (Fig. S23a), leading to the decrease in average charge of H^+^ to 0.35. In contrast, the presence of structural Al^3+^ in Al-MoO_x_ can significantly decrease the interaction between H^+^ and MoO_x_ with the enlarged electronic charge accumulation region around H^+^ (Fig. 3g). The Bader analysis also reveals the average charge of H^+^ is decreased to 0.25 (Fig. S23b). The weakened screening interaction induced by Al^3+^ introduction can promote the insertion of H^+^ into the electrode active material and thus improve the discharge/charge capacity. Meanwhile, the calculated total Gibbs free energy of protonated MoO_x_ for binding H^+^ ions decreases from -760.11 to -792.06 eV of Al-MoO_x_ (Table S5), which indicates the introduction of Al^3+^ can lower the Gibbs free energy of the ion-electrode system and thus enhance the capacity of electrodes [48]. The density of states (DOS) comparison of MoO_x_ and Al-MoO_x_ (Fig. 3h, i) shows the insertion of Al^3+^ leads to the remarkably increase in electron states near the Fermi level, leading to the appearance of metallic electronic properties [49, 50]. This transition can increase the electrical conductivity of the host material as well as its rate capabilities. This is consistent with the previous XPS result (Fig. 1b) that the electrons can be filled into the newly formed Mo (IV) in Al-MoO_x_, which causes the extension of occupied states toward Fermi level [51, 52].

**Fig. 4:**
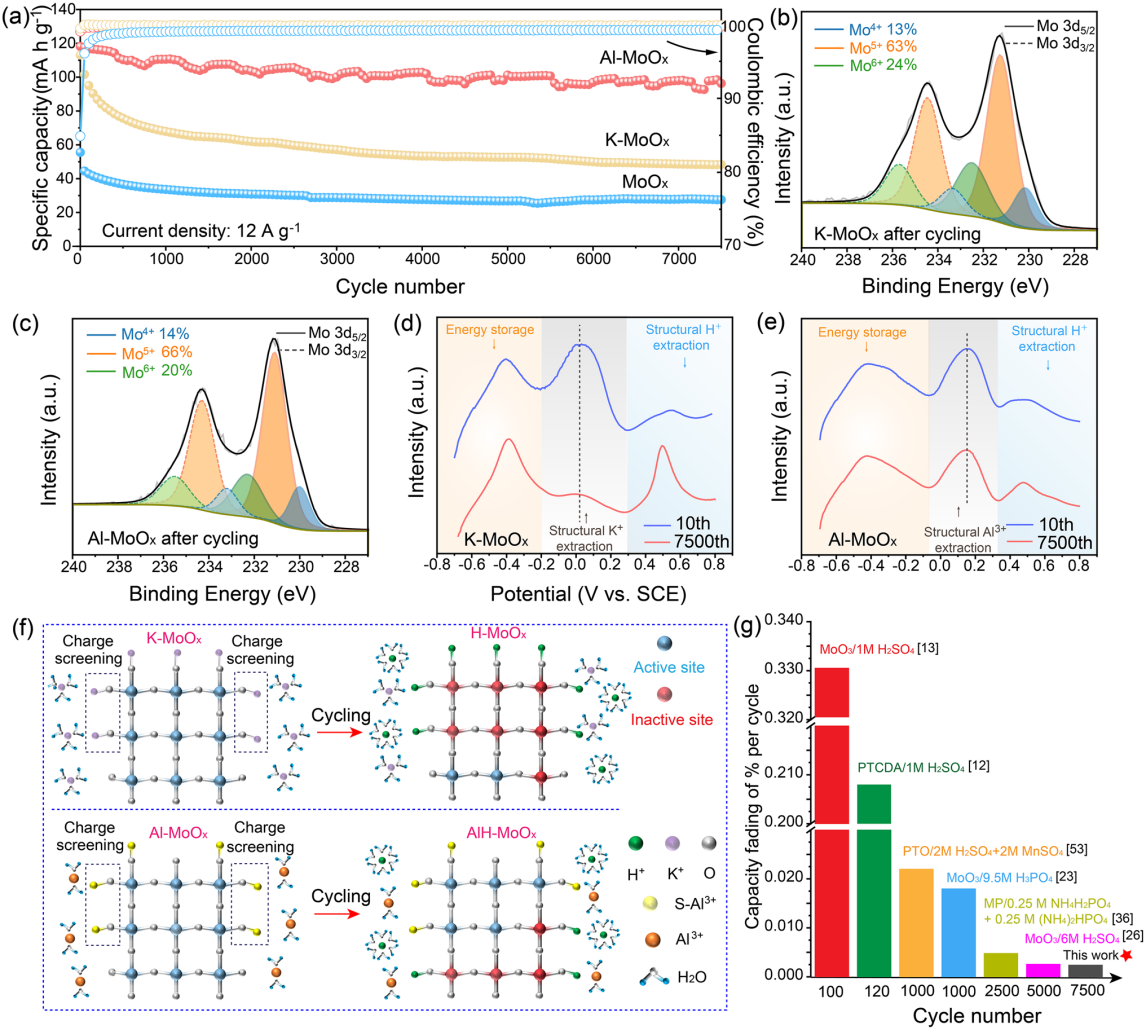
Analysis of high cyclic stability. **a** Cycling stability of MoO_x_, K-MoO_x_ and Al-MoO_x_. Mo 3d XPS spectra of **b** K-MoO_x_ and **c** Al-MoO_x_ after 7,500 cycles. LSV curves of structural cation extraction for **d** K-MoO_x_ and **e** Al-MoO_x_ before and after cycling. **f** Schematic illustration of energy storage mechanism of K-MoO_x_ and Al-MoO_x_ after cycling. **g** Cycling performance comparison between this work and the related studies

To further reveal the charge storage mechanism by exploring the influence of repeated H^+^ insertion/extraction on the structure of host materials, the cyclic stability of all samples was investigated by GCD measurement with the detailed result shown in Fig. 4a. Al-MoO_x_ can remain a reversible capacity of 96.3 mAh g^−1^ at 12 A g^−1^ after 7,500 cycles with the capacity retention of 81.2%, while MoO_x_ presented a very low value of 27.6 mAh g^−1^. In contrast, the capacity of K-MoO_x_ decays rapidly during the first 500 cycles and maintains only 42.8% of the initial capacity after 7,500 cycles, indicating that herein K^+^ can improve the capacity of MoO_x,_ while its inferior cycling stability limits its direct use as the anode material. The Al-MoO_x_ on the other hand can continuously work for more than 900 h at 1.2 A g^−1^ and deliver a capacity retention of 74.4% upon 2,000 GCD cycles (Fig. S24). Considering the low electrochemical activity and ultralow capacity of MoO_x_, we put the emphasis of further exploration on the electrochemical behaviors of Al-MoO_x_ and K-MoO_x_. The post-XRD measurements for the cycled electrodes show both electrodes can still remain the amorphous structure without crystalline phases formed (Fig. S25), suggesting that the difference in cyclic lifespans does not result from the formation of crystalline oxides. Different from the most reported cases of proton batteries in which performance attenuation is attributed to the electrode dissolution in acidic environment, the ICP-OES analysis shows nearly no Mo was observed in the electrolytes after long-term cycling (Table S6), demonstrating that other charge storage mechanisms responsible for the stability change of electrodes exist. By comparing the Mo states of M-MoO_x_ (M = Al, K) before and after cycling (Fig. 4b, c and Table S7), it can be clearly observed that the contents of Mo redox couples in K-MoO_x_ change significantly with the average charge of Mo decreased from 5.24 to 5.11. In contrast, the Mo oxidation state is stable with nearly no change for Al-MoO_x_ before and after long-term cycling. The subsequent elemental analysis shows the content of K^+^ within MoO_x_ is significantly reduced from 0.4 (K/Mo ratio) to 0.07 (Fig. S26a), demonstrating that most structural K^+^ has been substituted by the protons. Al^3+^ presented an excellent capability to resist the replacement from protons with only slight content change (0.15 vs. 0.11) even after 7,500 cycles (Fig. S26b).

To further elucidate the influence of proton exchange on the structure of electrode materials, LSV testing was performed on the electrodes for 7,500 cycles to examine the evolution of structural cations (Fig. 4d, e). The oxidation peak located between − 0.2 ~ 0.2 V is related to the extraction of structural metal ions (K: 0 V; Al: 0.16 V), while the peak at 0.5 V refers to structural H^+^ extraction. To quantitatively evaluate the proton replacement effect, the intensity ratio of peak height was calculated and denoted as *i*H/*i*M (M = Al, K) (Table S8). The plot shapes of Al-MoO_x_ before (running for 10 cycles for stabilization) and after long-term cycling are nearly no changed with very close *i*H/*i*Al values of 0.54 and 0.63 (Table S8). On the contrary, the peak related to structural K^+^ extraction nearly disappears after cycling (Fig. 4d) with dramatically enhanced intensity of irreversible H^+^ extraction peak. The *i*H/*i*K ratio is notably increased from 0.43 by > 5 times to 2.17, further confirming the serious proton exchange within K-MoO_x_. Note herein the different cycling performances of Al-MoO_x_ and K-MoO_x_ arise from the active material itself rather than the electrolytes since a control experiment for MoO_x_ in different electrolytes (AlCl_3_ and KCl with the same pH value) presents the similar capacity decaying (Fig. S27). The detailed energy storage mechanism is illustrated in Fig. 4f. The insertion/extraction of H^+^ during discharge/charge can induce the entry of partial irreversible proton into the lattice structure of MoO_x_, which leads to the originally active Mo atoms unavailable for charge storage (proton poisoning). The introduction of metal ions can effectively activate MoO_x_ by allowing more H^+^ insertion/extraction for reversible energy storage and elevating its capacity, while the accompanied more pronounced irreversible proton poisoning effect can be effectively suppressed by the presence of Al^3+^. Compared with monovalent K^+^, the trivalent Al^3+^ can have stronger ionic interactions with lattice oxygen for resisting their irreversible exchange with inserted metal ions and thus still maintain excellent structural stability after long-term cycling. Accordingly, the divalent Sr^2+^ introduced through the same procedure with that of Al^3+^ also presented the remarkable capability for improving the capacity while stabilizing the structure. Sr-MoO_x_ delivered a capacity of 89 mAh g^−1^ with an excellent retention rate of 79% after 7,500 cycles, much higher than that of K-MoO_x_ and slightly lower than that of Al-MoO_x_ (Figs. S28 and S29). In addition, the cycling performance of Al-MoO_x_ was investigated in HCl electrolyte (Fig. S30a). Compared with AlCl_3_ electrolyte, the result shows Al^3+^ in the electrolyte can effectively improve the cycling stability of Al-MoO_x_. The elemental analysis shows the content of Al^3+^ within MoO_x_ is significantly reduced from 0.15 (Al/Mo ratio) to 0.06 (Fig. S30b). This result shows Al^3+^ in electrolyte is helpful to stabilize the composition of Al-MoO_x_, resulting in the improvement of cycling stability. Furthermore, the performance of crystalline MoO_3_ was also evaluated and it can only deliver a low capacity of 56 mAh g^−1^ with a retention of only 28% even after 2,000 cycles (Fig. S31). Figure 4g lists the long-term cycling performance position of Al-MoO_x_ among reported peer electrodes for hydrogen storage and its capacity fading of 0.0024% per cycle outperforms nearly all of the state-of-the-art counterparts (Table S9) [12, 13, 23, 26, 53].

### Electrochemical Performances of the Full Cell

**Fig. 5:**
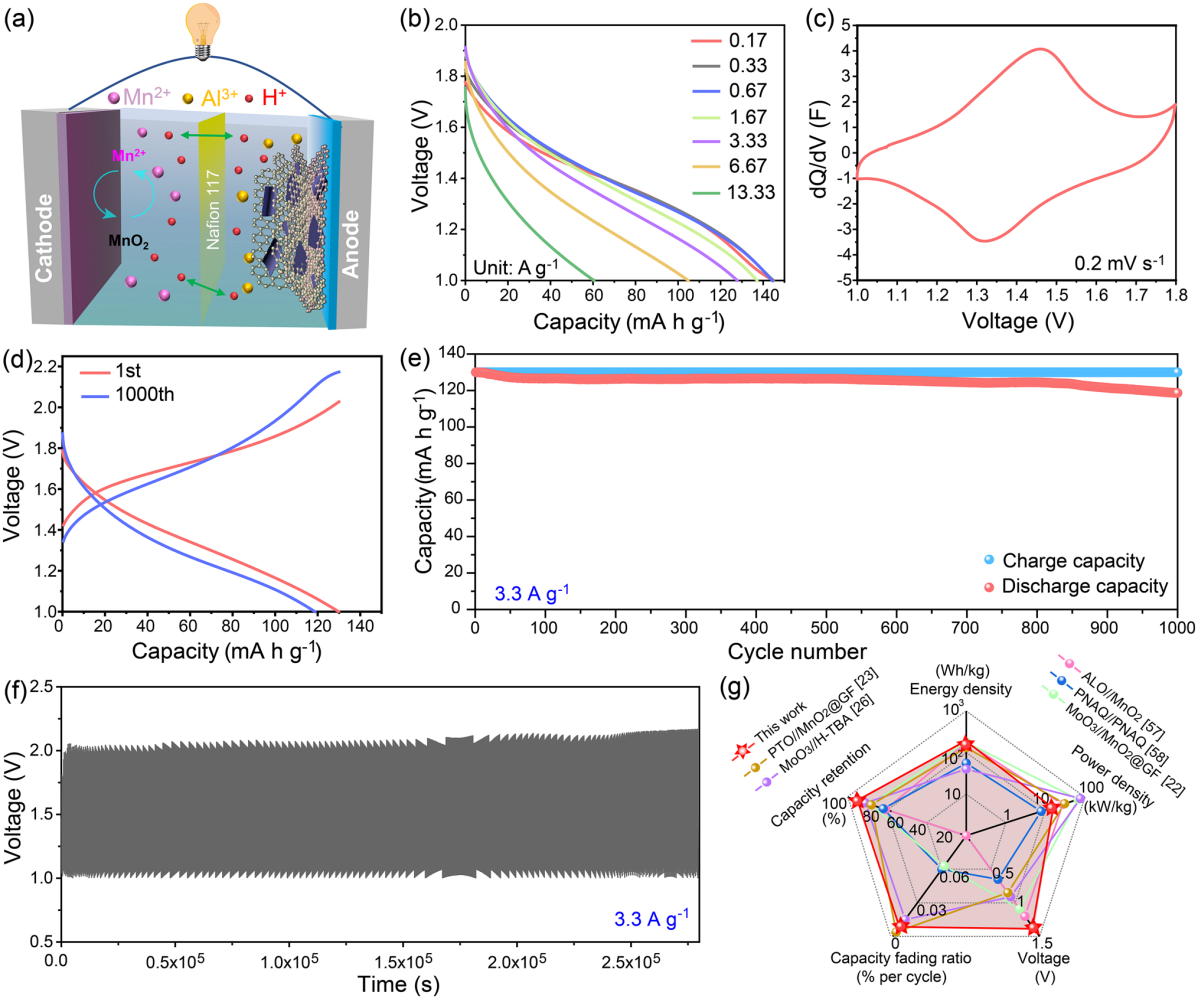
Full-cell performances. The battery was assembled with Al-MoO_x_ as the anode and the commonly used MnO_2_ as the cathode. **a** Schematic illustration of battery assembly and the corresponding energy storage process. **b** Discharge profiles at different current densities. **c** The associated differential capacity curve at 0.2 mV s^−1^. **d** GCD profile comparison at the 1st and the 1000th cycles. **e** Cycling performance at 3.3 A g^−1^. **f** Voltage–time curve after 1000 cycles. **g** Performance comparison between this work and reported studies with radar chart including energy density, power density, voltage, capacity fading ratio and capacity retention

A H-type proton full cell was assembled using Al-MoO_x_ as the anode and MnO_2_ as the cathode (Fig. 5a). Nafion 117 proton exchange membrane was used as the separator and H^+^ served as the charge carrier. Structural characterization and electrochemical performances of MnO_2_ were investigated with the result shown in Fig. S32. The anodic chemistry is carried out by H^+^ and less Al^3+^ insertion/extraction in Al-MoO_x_ (Eq. 6), while the cathodic chemistry is dependent on the MnO_2_/Mn^2+^ two-phase conversion (Eq. 7).

7 $${\text{Mn}}^{{2 + }} {\text{ + 2H}}_{{2}} {\text{O}} \leftrightarrow {\text{MnO}}_{{2}} {\text{ + 2e}}^{ - } {\text{ + 4H}}^{ + }$$

The GCD profiles at different current densities show the full cell can deliver a high discharge capacity of 145 mAh g^−1^ at 0.17 A g^−1^ while still maintaining 60.4 mAh g^−1^ when the current density was increased by nearly 80 times to 13.3 A g^−1^, demonstrating its fast kinetics for hydrogen storage (Fig. 5b). A pair of redox peaks with small spacing can be observed in its CV curve at 0.2 mV s^−1^, which indicates its excellent electrochemical reversibility. More importantly, a high average voltage of 1.37 V is achieved for the full cell, which surpasses most reported proton batteries (Fig. 5c) [54]. Correspondingly, a high energy density of 160.2 Wh kg^−1^ at a power density of 184.3 W kg^−1^ can be delivered, which is among the highest values of the reported aqueous proton batteries (Table S10). Note herein the high voltage is not only important for the improvement of energy density, but also is crucial for the choice of electrode materials. This high-voltage window allows the further optimization of full-cell configurations in the future work, which may bring the potential economic benefits for commercialization of PBs. Meanwhile, it also can retain a high power density of 13.4 kW kg^−1^ at an energy density of 60.7 Wh kg^−1^, which is comparable to the state-of-the-art proton batteries and zinc-ion batteries [54–56]. The assembled device also presented excellent cyclic performance with the capacity retention rate of 91.3% after 1,000 cycles, while the capacity fading rate per cycle is only 0.0087% at 3.3 A g^−1^ (Fig. 5d, e). The upper limit of the cell voltage shows a very slight change from 2.03 to 2.17 V, indicating that the electrode polarization is negligible in the cycling (Fig. 5f). A radar diagram of the important full-cell metrics including energy density, power density, average voltage, capacity retention rate and capacity fading rate is plotted in Fig. 5g to provide the all-sided view for evaluating the performance of batteries [22, 23, 26, 57, 58]. The balanced properties of Al-MoO_x_-based full cell that surpass its state-of-the-art PB counterparts demonstrate its great promise for the next-generation hydrogen storage device (Table S10).

## Conclusion

In this work, we show amorphous electrodes of proton batteries by proposing a general ion-exchange strategy for activating MoO_x_ with inserted multivalent metal cations. Taking Al^3+^ as an example, the pre-inserted K^+^ can weaken the interaction between Al^3+^ and lattice oxygen, leading to a substantial increase in Al^3+^ content within MoO_x_ that cannot be achieved by conventional direct insertion. The as-prepared Al-MoO_x_ shows a different charge storage behavior from MoO_x,_ while the presence of Al^3+^ can effectively shield the electrostatic interaction between hydrogen ions and lattice oxygen, endowing the electrode with high capacity and rate capability. Meanwhile, the performance fading of molybdenum oxide caused by the insertion of excessive structural hydrogen ions can be remarkably suppressed by Al^3+^, and thus, Al-MoO_x_ achieved the superior capacity retention of 81.5% after 7,500 cycles. The full cell assembled from Al-MoO_x_-based anode and MnO_2_ cathode delivered a high average voltage of 1.37 V, which exceeds most reported proton batteries. A high energy density of 160.2 Wh kg^−1^ at a power density of 184.3 W kg^−1^ with outstanding cyclic stability was also achieved. This work is expected to trigger a new material system for proton batteries and may potentially shed light on electrode design for broad energy storage devices.

### Supplementary Information

Below is the link to the electronic supplementary material.

Supplementary file1 (PDF 3690 KB)
