# Supplementary material for: An Amorphous Anode for Proton Battery
Source: Nanomicro Lett. 2022 Dec 30;15:24. doi: 10.1007/s40820-022-00987-2 (PMC9803796; doi:10.1007/s40820-022-00987-2)
Supplement: Supplementary file 1 — Supplementary file1 (PDF 3690 KB) [file 40820_2022_987_MOESM1_ESM.pdf]

Supporting Information for

An Amorphous Anode for Proton Battery

Huan Liu[1], Xiang Cai[2], Xiaojuan Zhi[1, 3], Shuanlong Di[1], Boyin Zhai[1], Hongguan Li[3, 4], Shulan Wang[1], and Li Li[3, 4, *]

[1] Department of Chemistry, College of Science, Northeastern University, Shenyang 110819, Liaoning, P. R. China

[2] School of Light Industry and Chemical Engineering, Dalian Polytechnic University, Dalian 116034, Liaoning, P. R. China

[3] State Key Laboratory of Rolling and Automation, Northeastern University, Shenyang 110819, Liaoning, P. R. China

[4] School of Metallurgy, Northeastern University, Shenyang 110819, P. R. China

* Corresponding author. E-mail: lilicmu@alumni.cmu.edu (Li Li)

Supplementary Figures and Tables

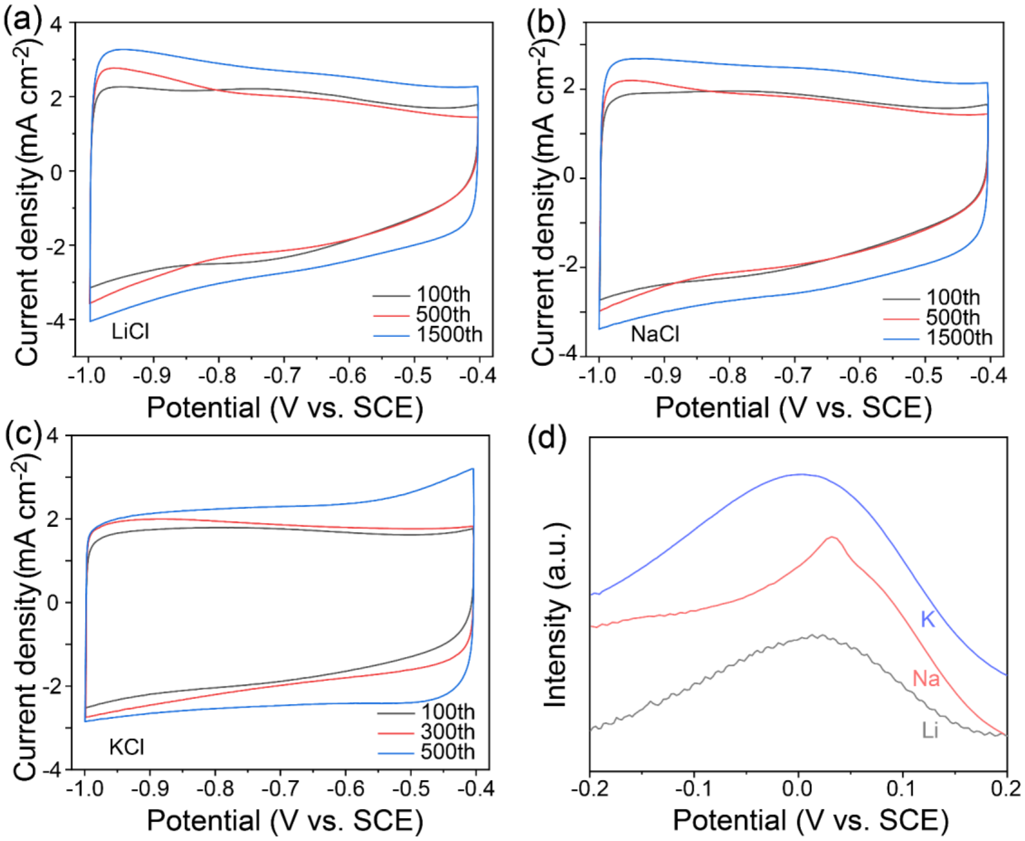

Fig. S1 CV curves of MoO_x in **a** LiCl, **b** NaCl, and **c** KCl electrolytes at 5 mV s^{-1}. **d** LSV curves of structural cation extraction for M-MoO_x (M = Li, Na and K)

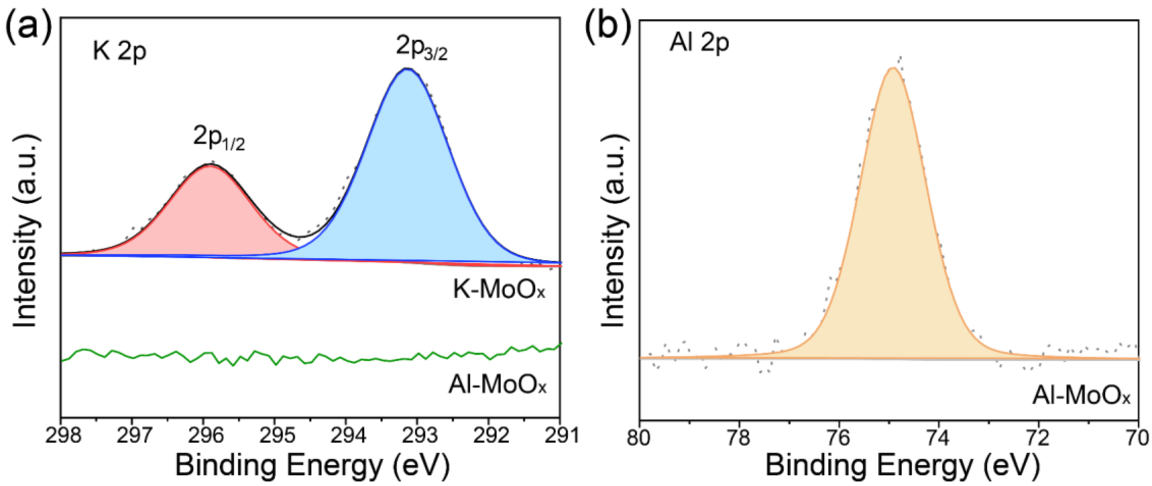

Fig. S2 a K 2p XPS spectra of K-MoO_x and Al-MoO_x. **b** Al 2p XPS spectra of Al-MoO_x

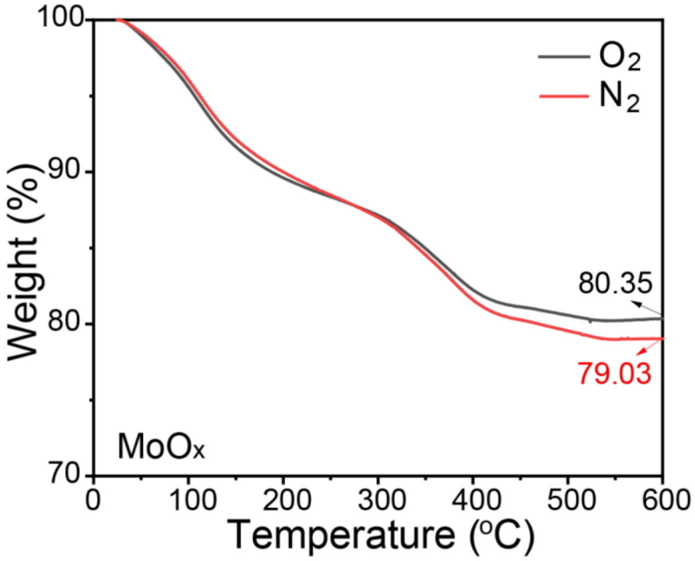

Fig. S3 TGA analysis of MoO_x in air and nitrogen atmosphere with a heating rate of 10 ^oC/min

The MoO_x sample was analyzed by the thermogravimetric analysis (TGA) under nitrogen and oxygen atmospheres to determine the value of x [S1]. With the increase of heating temperature, the adsorbed and structural water within MoO_x can be released and the sample is fully converted into MoO_3 at around 600 ^oC. Equation S1 shows the calculation of the oxygen vacancy concentration based on TGA analysis:

$$\frac{M(MoO_3)}{M(Mo)+xM(O)} = \frac{80.35\%}{79.03\%} \qquad (S1)$$

The molecular formula of MoO_x obtained from Eq. S1 is $MoO_{2.85}$, and the oxygen vacancy concentration is calculated to be 0.15.

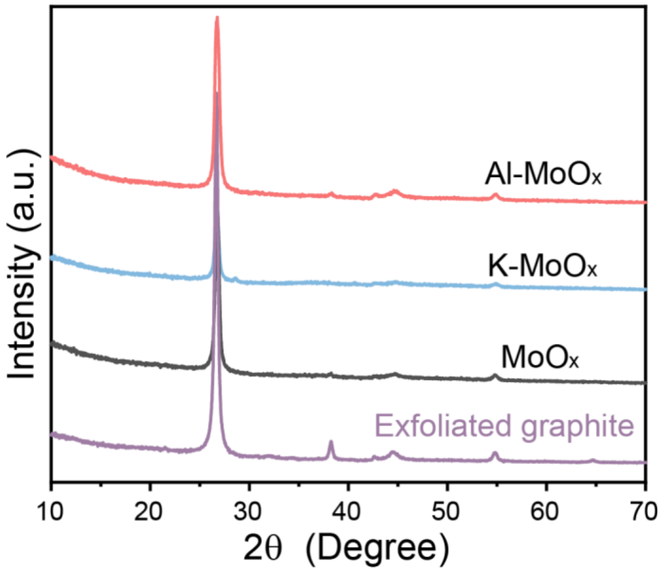

Fig. S4 XRD patterns of electrodes

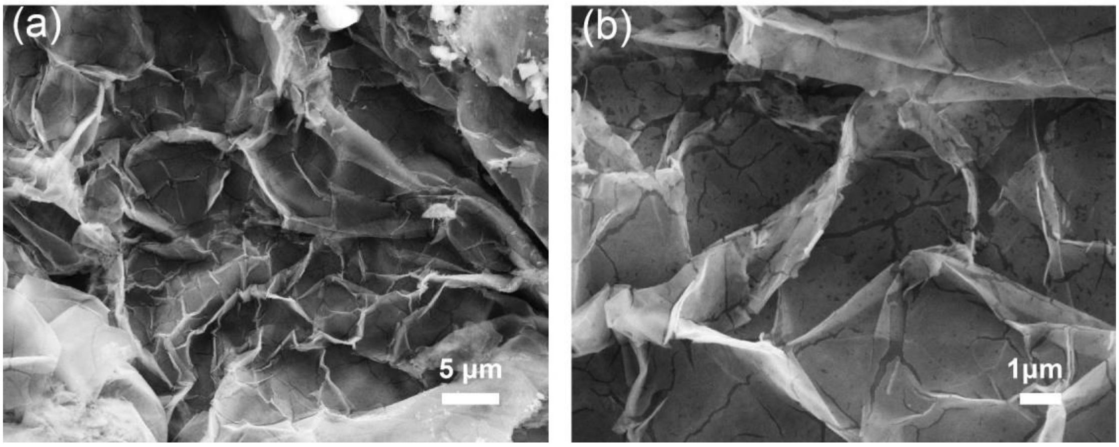

Fig. S5 SEM images of MoO_x at different magnifications

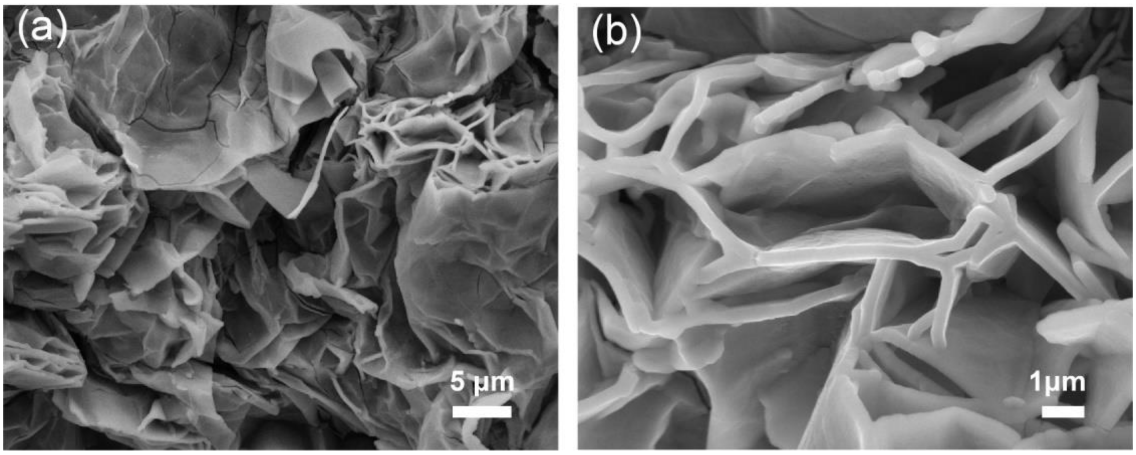

Fig. S6 SEM images of K-MoO_x at different magnifications

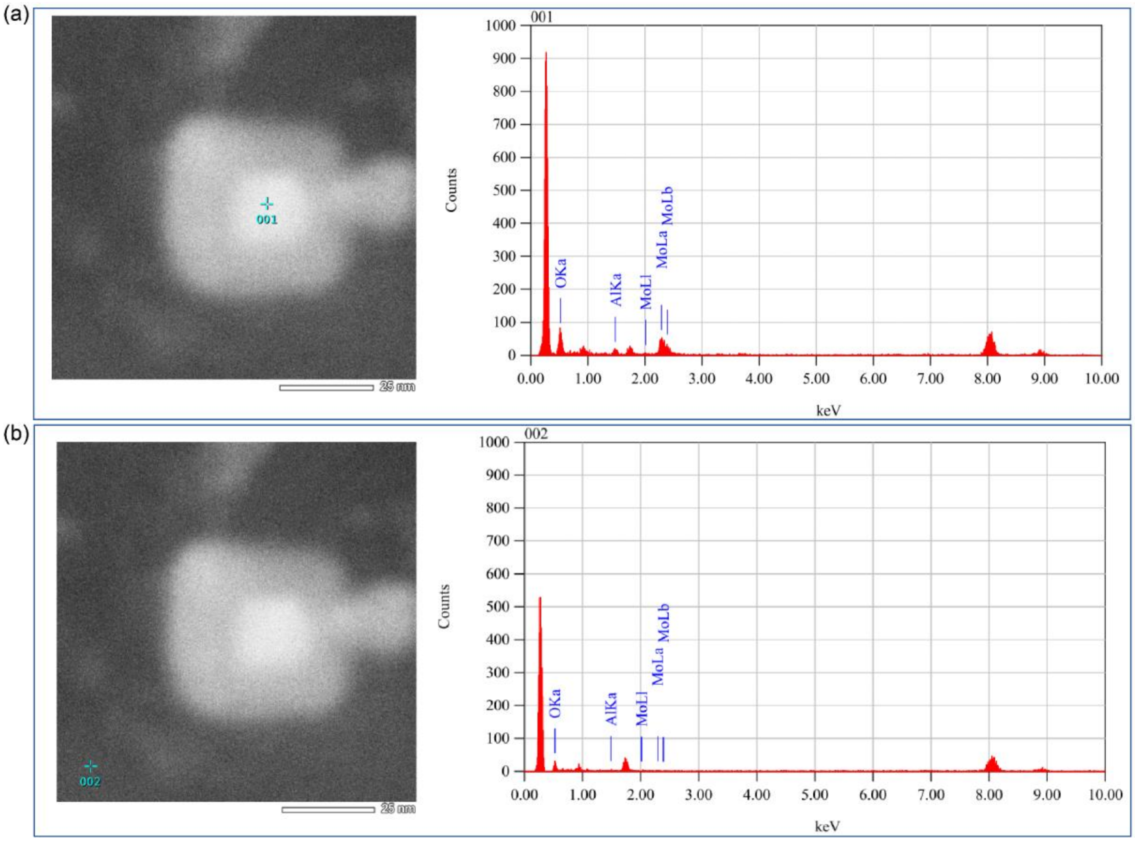

Fig. S7 EDS results of **a** Al-MoO_x and **b** its adjacent region

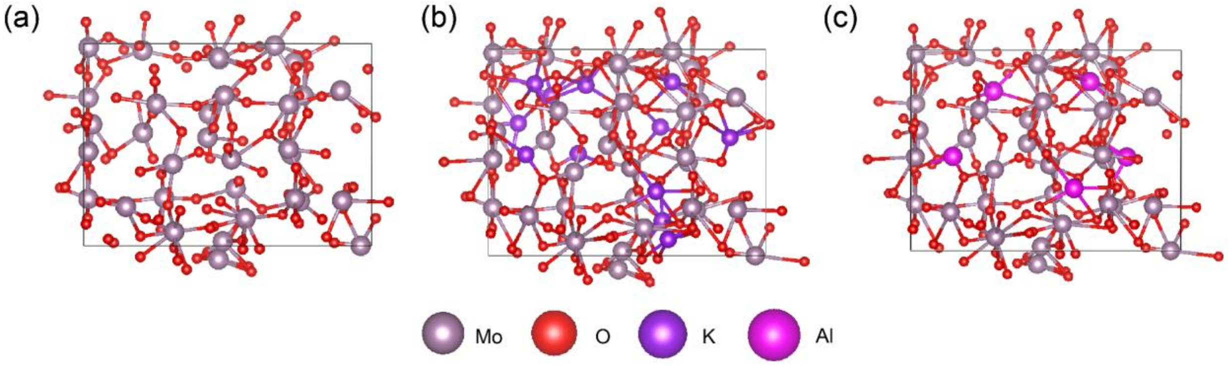

Fig. S8 DFT-optimized structures of **a** MoO_x, **b** K-MoO_x and **c** Al-MoO_x

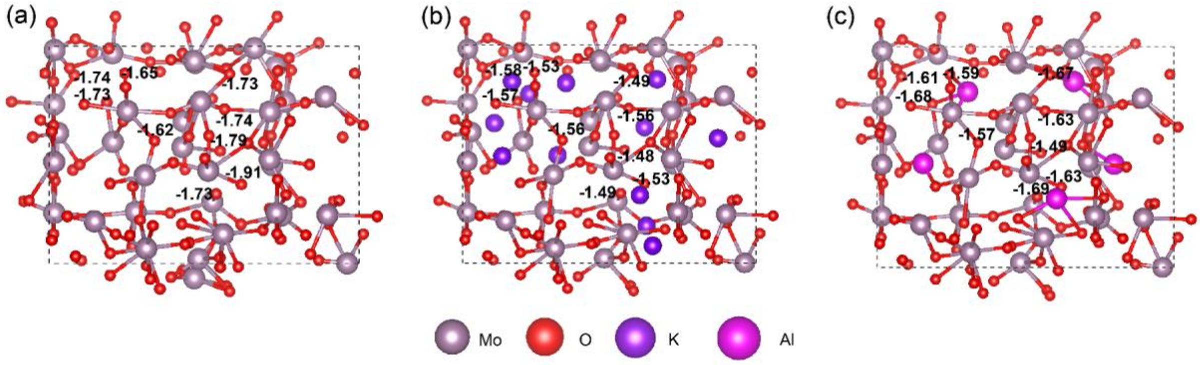

Fig. S9 Bader charge of **a** MoO_x, **b** K-MoO_x and **c** Al-MoO_x

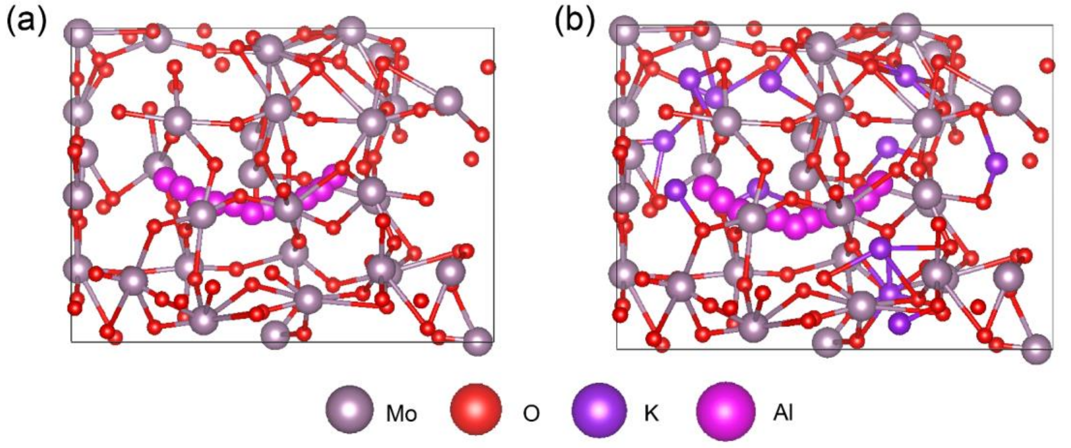

Fig. S10 The migration path of Al^{3+} in **a** MoO_x and **b** K-MoO_x

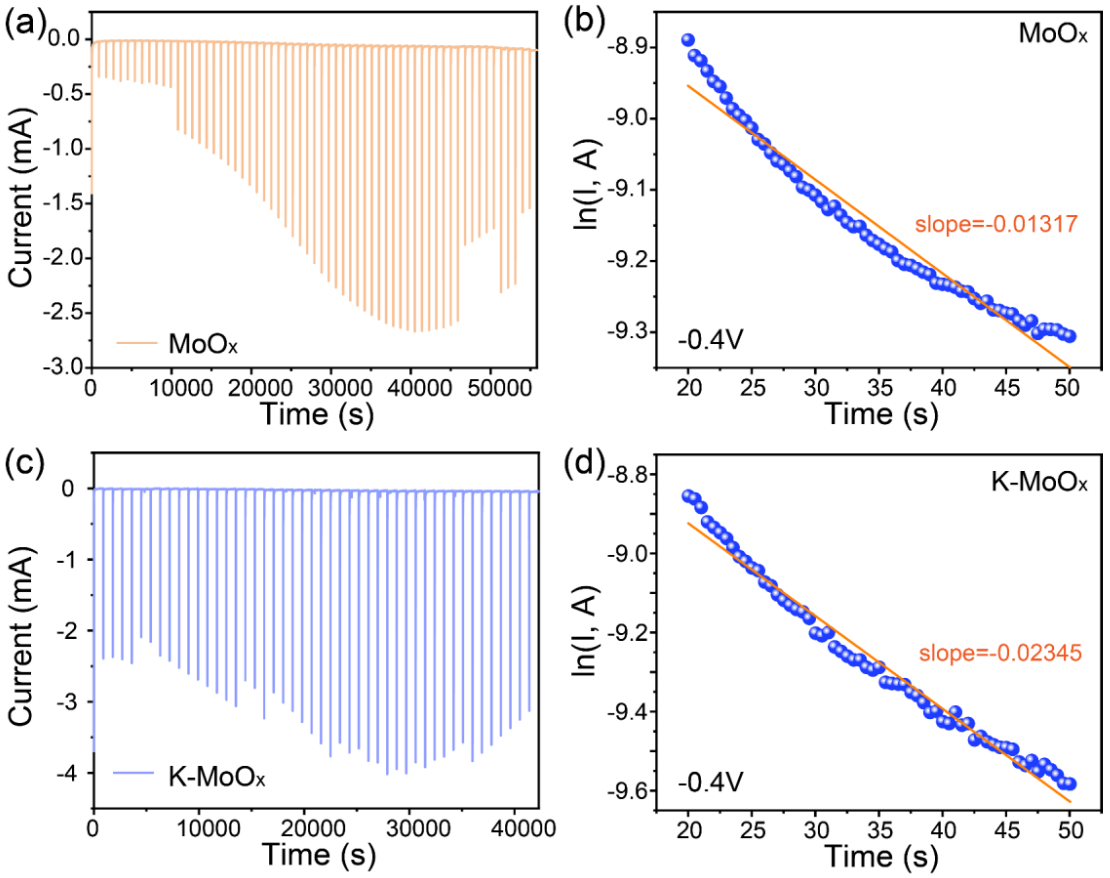

Fig. S11 PITT profiles as well as linear fitting of lnI-t slope for **a**, **b** MoO_x and **c**, **d** K-MoO_x

The corresponding co-insertion of Al^{3+} and H^+ diffusion coefficients of K-MoO_x and MoO_x were calculated according to Eq. S2:

$$D = -\frac{d\ln I(t)}{dt}\frac{4L^2}{\pi^2} \qquad \text{(S2)}$$

where D is chemical diffusion coefficient, L the electrode thickness, I the current and t the time.

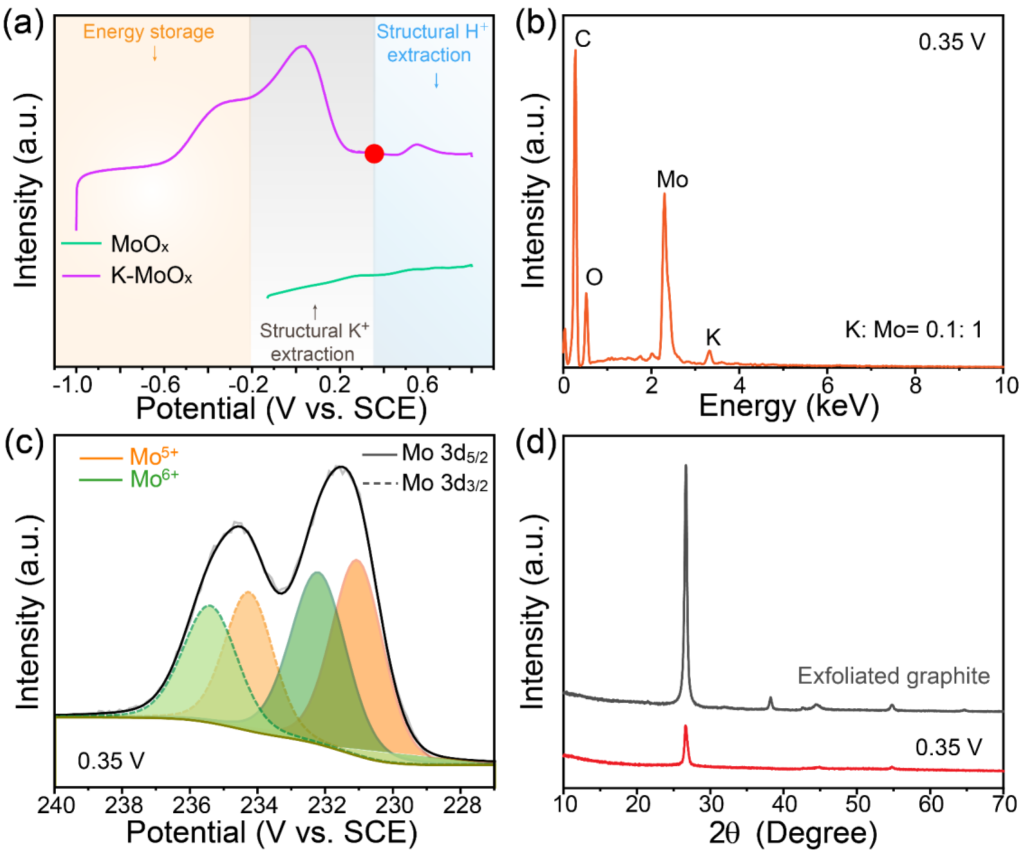

Fig. S12 a LSV curves of MoO_x and K-MoO_x in 2 M KCl at 0.5 mV s^{-1}. **b** EDS spectrum, **c** Mo 3d XPS spectrum and **d** XRD pattern of the sample at red dot (0.35 V) in (a)

To further prove that the oxidation peak around 0 V at Fig. 4d corresponds to the extraction of structural K^+, the LSV curves of MoO_x before and after CV running at 2 M KCl was compared (Fig. S12a). It can be clearly observed that no peak was observed for MoO_x without K^+ insertion (green curve) while the peak appears for the K-MoO_x between 0 V and 0.35 V. The detailed EDX analysis (Fig. S12b) for the sample obtained at 0.35 V confirms the remaining of K to Mo is 0.1:1 after extraction while XPS result shows the average valence of Mo also increased from 5.24 to 5.49 due to the extraction of structural K^+ in K-MoO_x. The corresponding XRD result indicates no crystalline MoO_3 was formed during the process, illustrating that the increase in the average valence of Mo is caused by the removal of structural K^+.

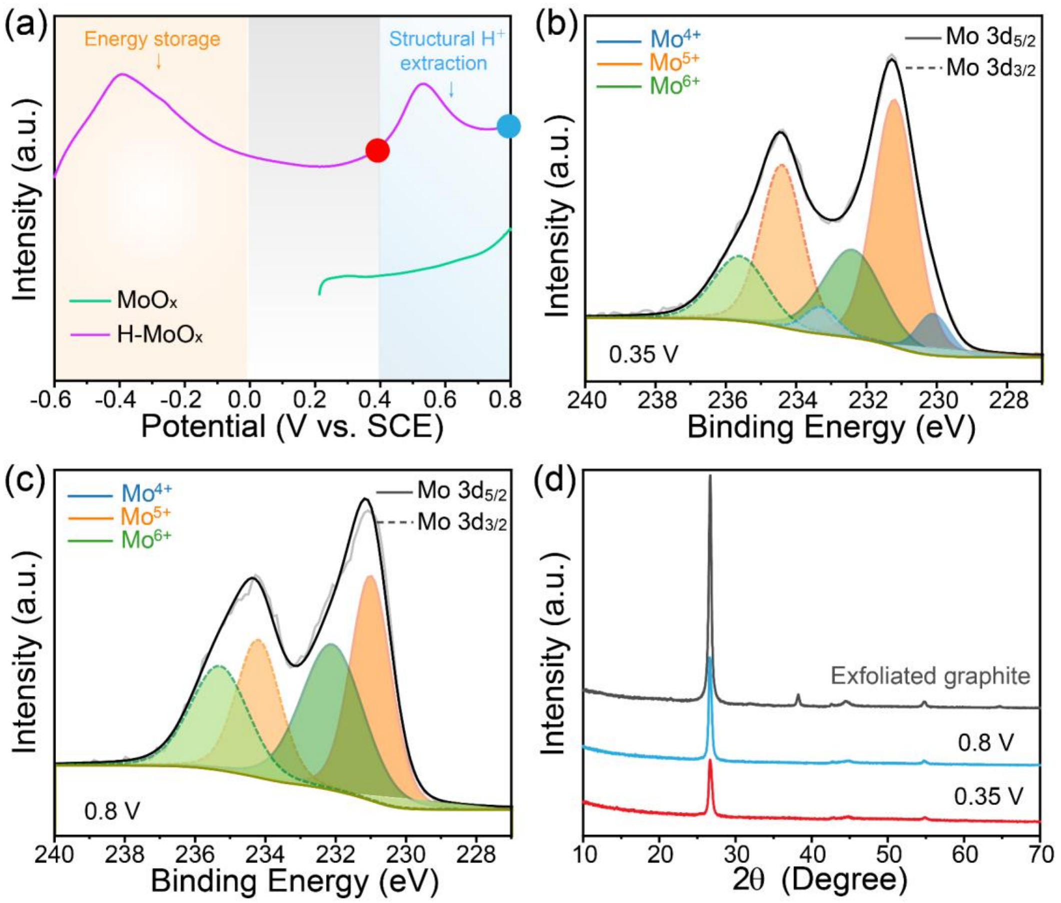

Fig. S13 a LSV curves of MoO_x and H-MoO_x in HCl (with the same pH to that of 2 M $AlCl_3$) at 0.5 mV s^{-1}. Mo 3d XPS spectra of the sample obtained at **b** 0.35V (red dot) and **c** 0.8V (blue dot) as well as **d** the corresponding XRD patterns in (a)

To prove that the oxidation peak of 0.5 V corresponds to the extraction of structural H^+ in Fig. 4d, the LSV curves of MoO_x before and after CV running in HCl (H-MoO_x) with a pH value of 0.4 (the same pH value to $AlCl_3$ used for Al-MoO_x) were compared in Fig. S13a. It can be clearly observed that a peak at around 0.5 V appears for H-MoO_x while no similar peak is observed for MoO_x. Correspondingly, the peak at 0 V that is related to the extraction of structural K^+ is absent for H-MoO_x. The subsequent XPS analysis for the two samples obtained at 0.35 V and 0.8 V indicates the valence state of Mo increases from 5.23 (at 0.35 V) to 5.5 (at 0.8 V) because of the structural H^+ extraction. XRD analysis did not detect the formation of any crystalline phase, which also confirms the increase of Mo valence state is attributed to H^+ extraction.

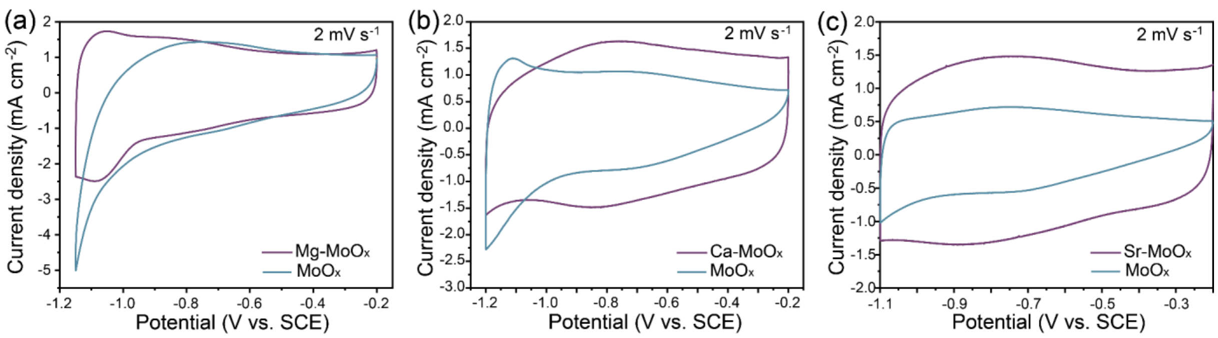

Fig. S14 CV curves of **a** Mg-MoO_x and MoO_x, **b** Ca-MoO_x and MoO_x, and **c** Sr-MoO_x and MoO_x at 2 mV s^{-1} in the corresponding electrolytes

It can be clearly observed that the CV area of M-MoO_x (M= Mg, Ca, Sr) is apparently larger than that of MoO_x, respectively, confirming the enhanced capacity of M-MoO_x with effectively inhibited hydrogen evolution reaction.

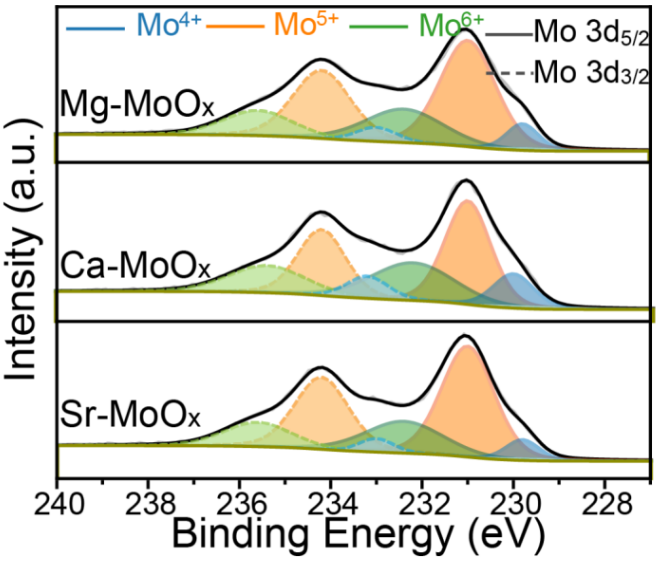

Fig. S15 Mo 3d XPS spectra of Mg-MoO_x, Ca-MoO_x, and Sr-MoO_x

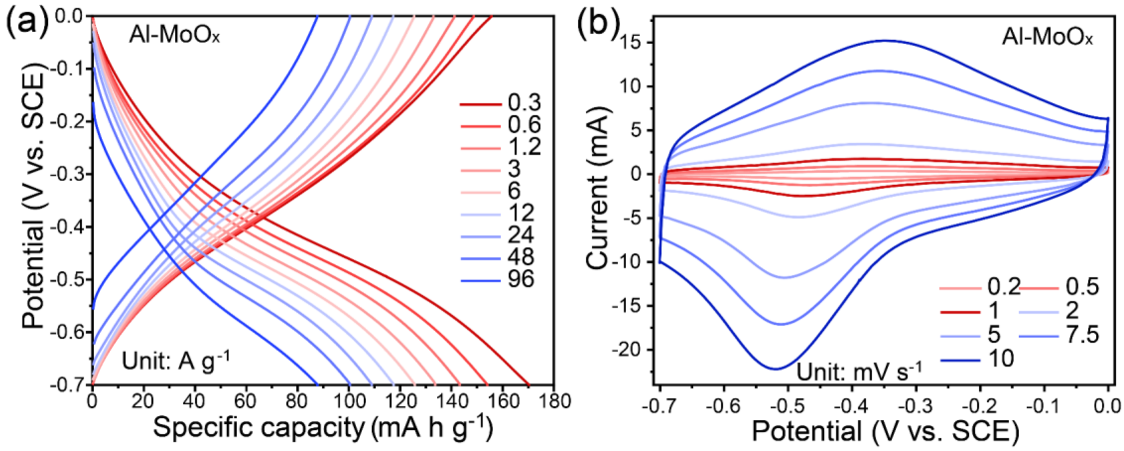

Fig. S16 a GCD curves of Al-MoO_x at different current densities. **b** CV curves of Al-MoO_x at different scan rates

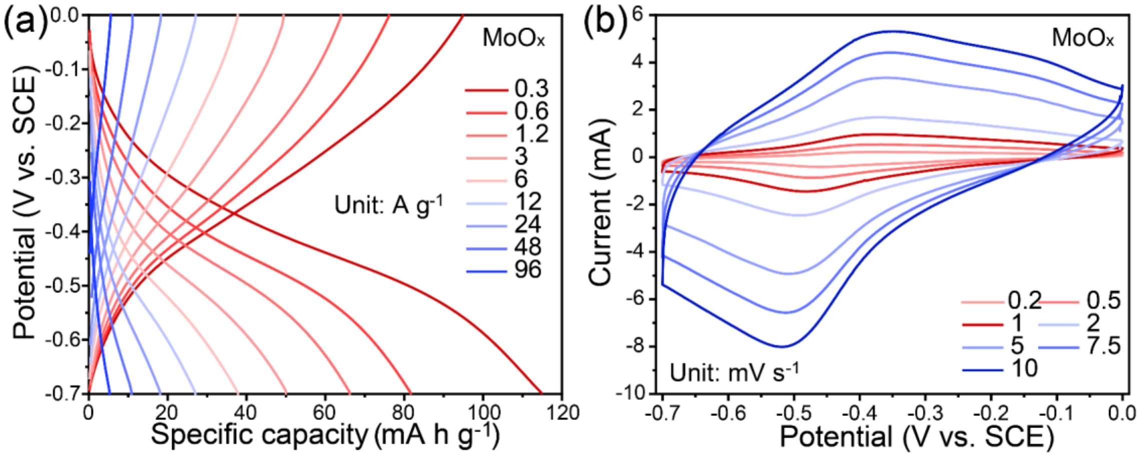

Fig. S17 a GCD curves of MoO_x at different current densities. **b** CV curves of MoO_x at different scan rates

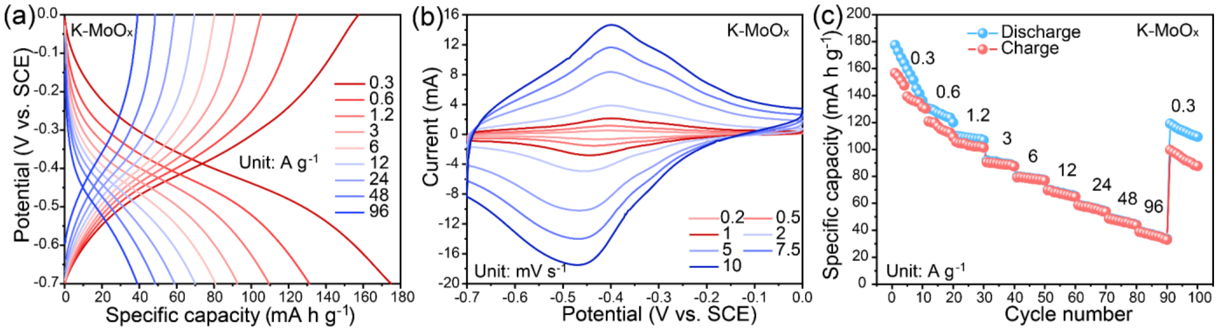

Fig. S18 a GCD curves of K-MoO_x at different current densities. **b** CV curves of K-MoO_x at different scan rates. **c** Rate capability of K-MoO_x

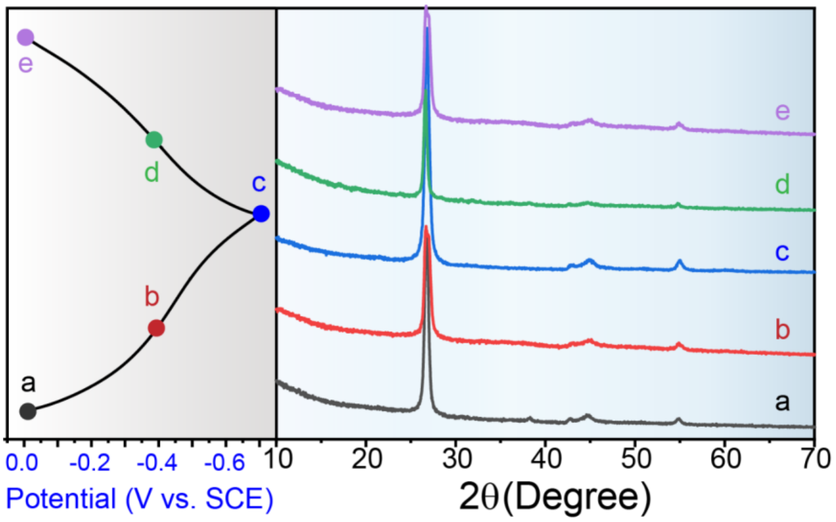

Fig. S19 XRD patterns of Al-MoO_x at different states of charge

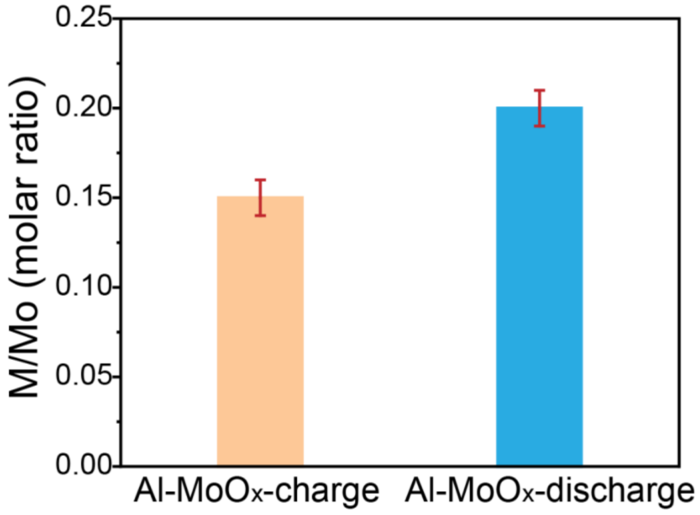

Fig. S20 ICP-OES results of Al-MoO_x at charge/discharge states

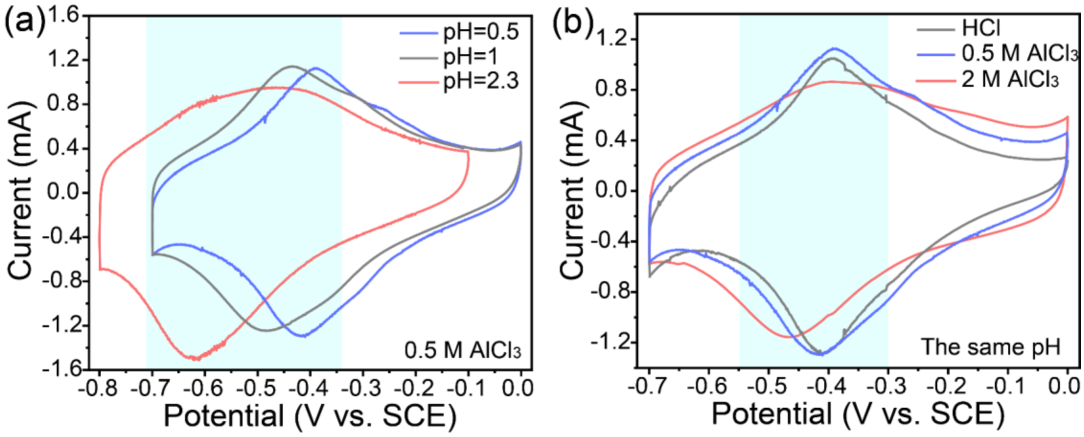

Fig. S21 CV curves of **a** Al-MoO_x in 0.5 M $AlCl_3$ electrolyte with different pH values adjusted by the addition of HCl, and **b** Al-MoO_x in different electrolytes with the same pH (the pH value of 0.5 M $AlCl_3$ was adjusted by the addition of HCl)

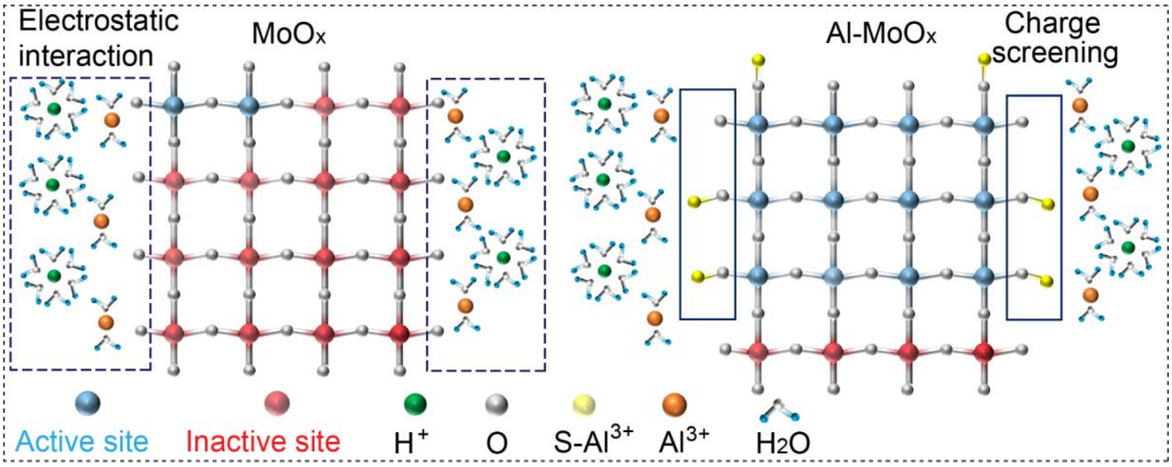

Fig. S22 Schematic illustration of the energy storage mechanism of MoO_x and Al-MoO_x. The presence of Al^{3+} can weaken the interactions between structural O and inserted H^+, promoting the insertion of H^+ into the host MoO_x

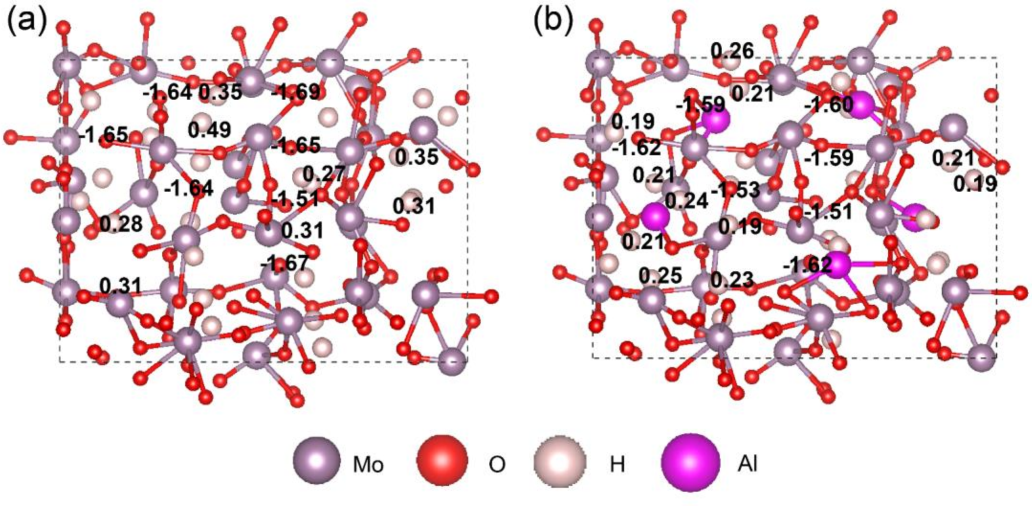

Fig. S23 Bader charge of protonated **a** MoO_x and **b** Al-MoO_x

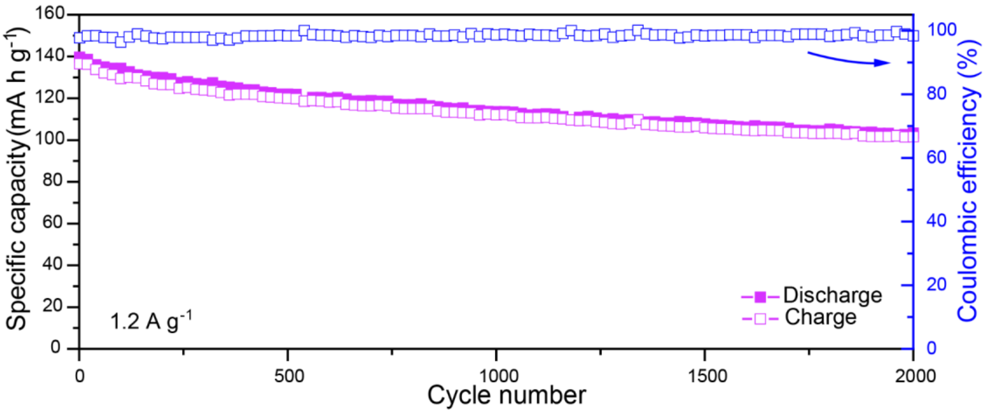

Fig. S24 Cycling performance of Al-MoO_x at 1.2 A g^{-1}

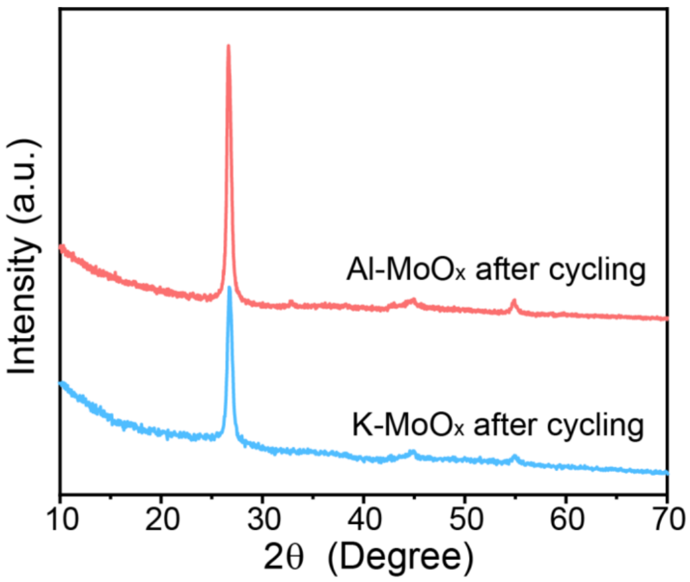

Fig. S25 XRD patterns of Al-MoO_x and K-MoO_x after 7500 cycles

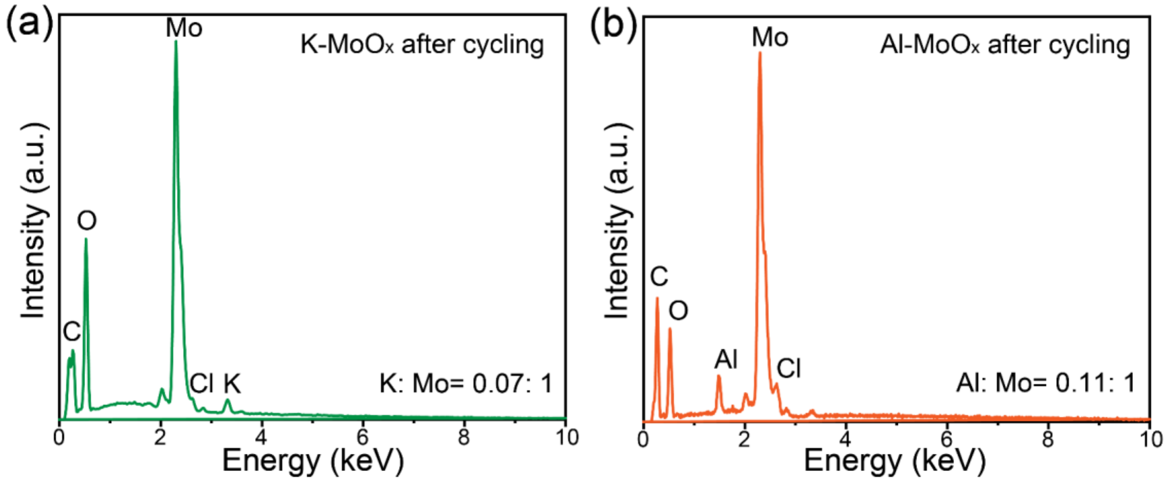

Fig. S26 EDS spectra of **a** K-MoO_x and **b** Al-MoO_x after 7500 cycles

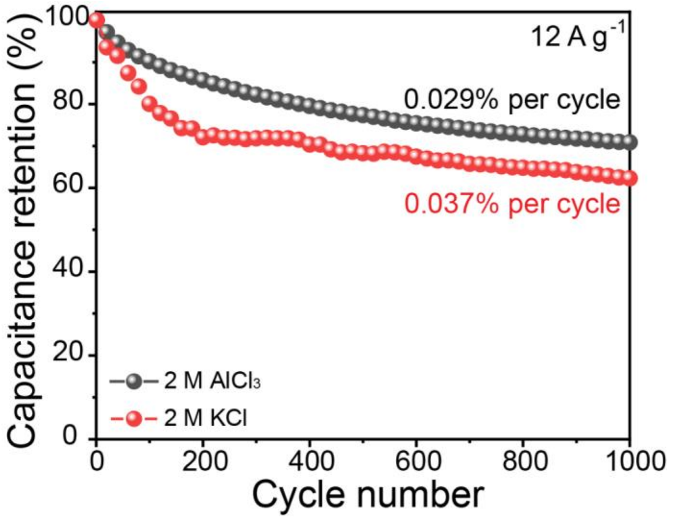

Fig. S27 Cycling performances of MoO_x in the electrolytes of 2 M $AlCl_3$ and 2 M KCl (with the addition of HCl to adjust its pH value to be the same with that of 2M $AlCl_3$) at 12 A g^{-1}

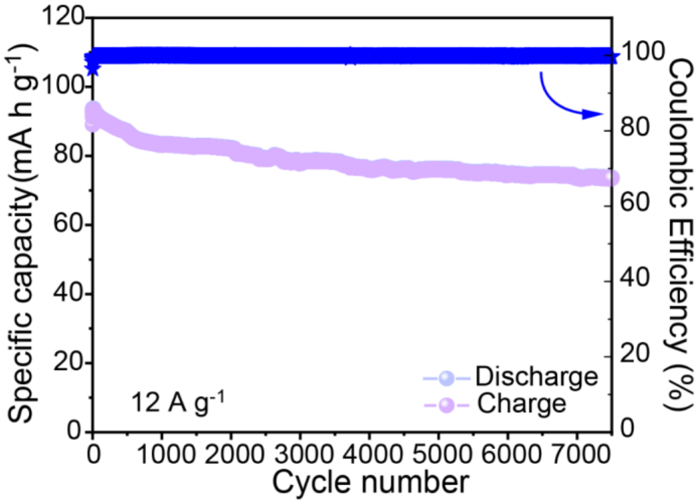

Fig. S28 Cycling performance of Sr-MoO_x in 2 M $SrCl_2$ (with the addition of HCl to adjust its pH value to be the same with that of 2 M $AlCl_3$) at 12 A g^{-1}

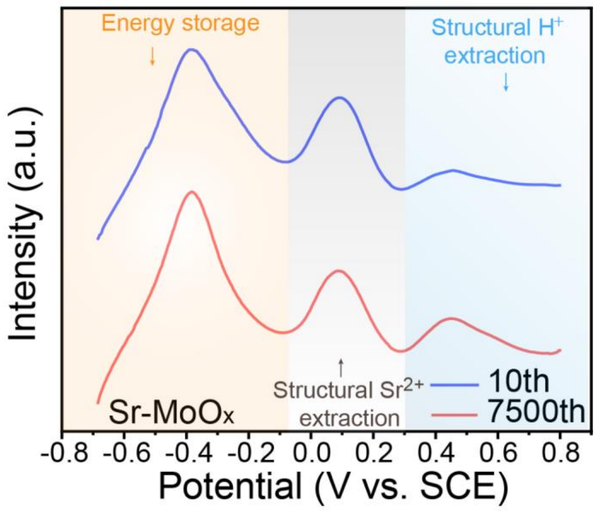

Fig. S29 CV curves of structure cation extraction for Sr-MoO_x before and after cycling. The shape of CV curves is not changed and the corresponding iH/iSr values are close (0.49 -10th cycle vs. 0.65-7500th cycle)

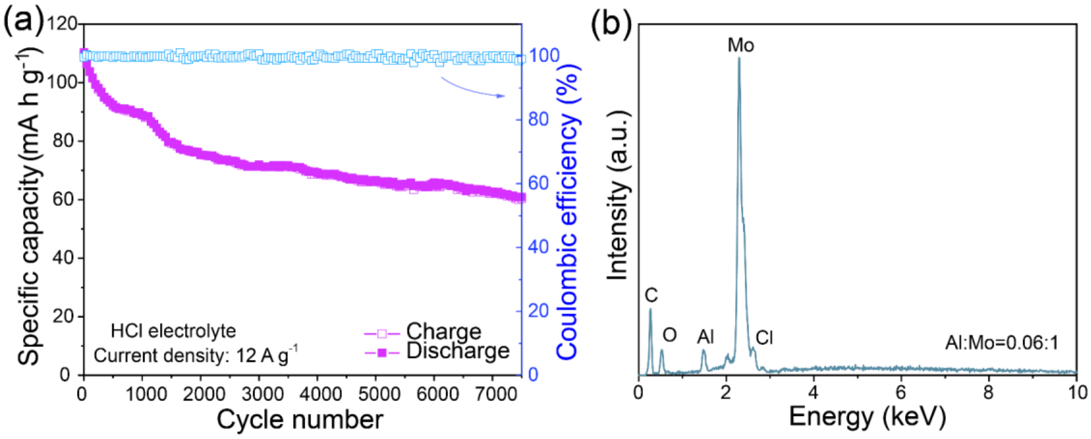

Fig. S30 a Cycling performance of Al-MoO_x at 1.2 A g^{-1} and **b** EDS spectra of Al-MoO_x after 7500 cycles in HCl electrolyte (adjusted with the same pH value to that of 2 M $AlCl_3$)

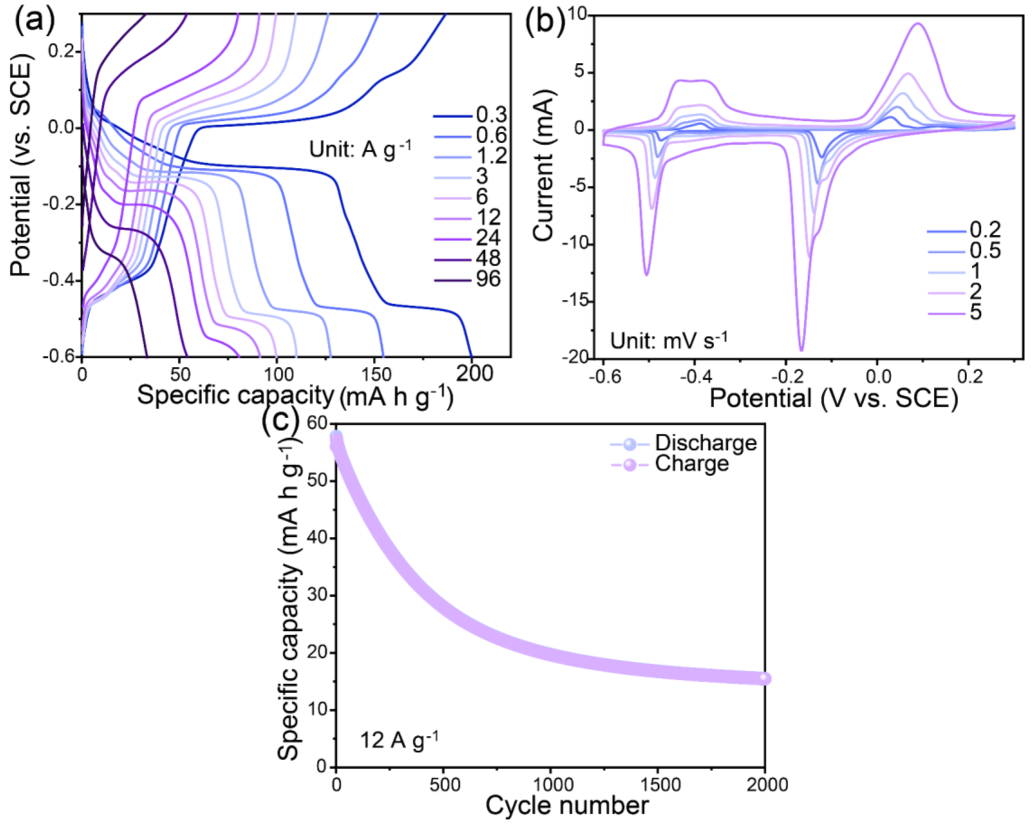

Fig. S31 Electrochemical performances of crystalline MoO_3 in 2 M $AlCl_3$: **a** GCD curves at different current densities. **b** CV curves at different scan rates. **c** Cycling performance at 12 A g^{-1}

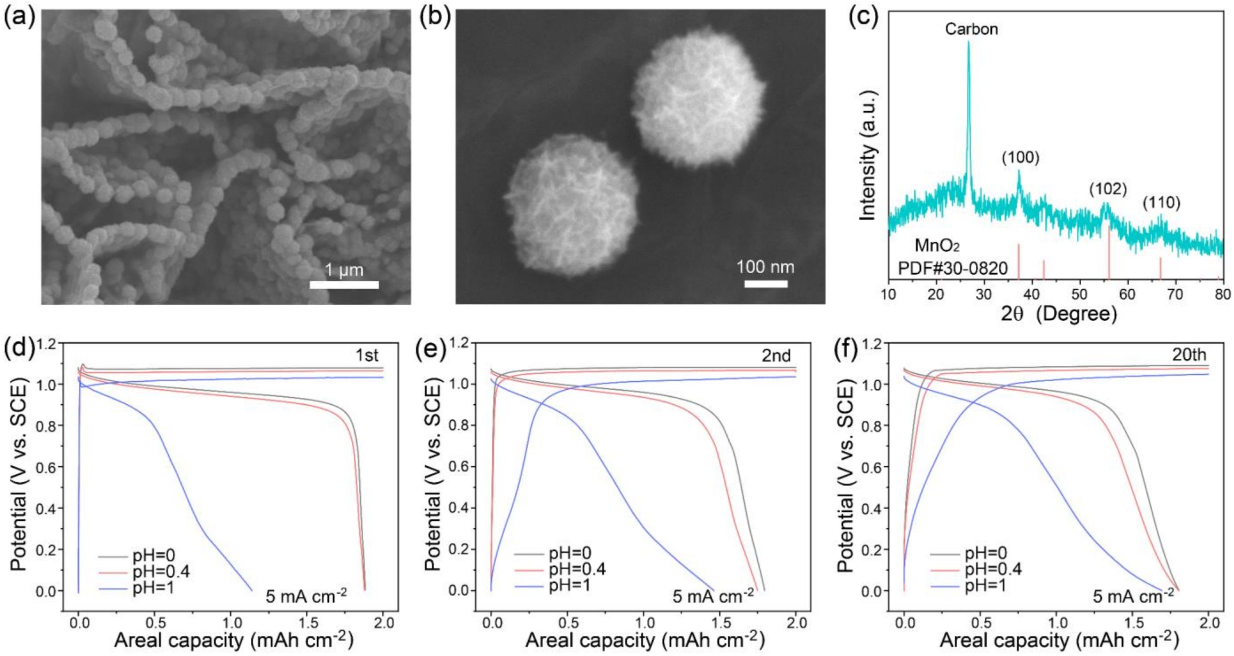

Fig. S32 Structural characterizations and electrochemical performances of MnO_2. **a, b** SEM images, and **c** XRD patterns of MnO_2. Galvanostatic discharge-charge profiles of EG electrode in different electrolytes at **d** the first, **e** the second, and **f** the 20th cycling

Table S1 Mo 3d XPS results of the as-prepared MoO_x, K-MoO_x and Al-MoO_x

Samples	Component (eV)	Content (%)	Average valence	Assignment
MoO_x	231.1	40	5.6	$Mo^{5+}(3d_{5/2})$
	234.3			$Mo^{5+}(3d_{3/2})$
	232.8	60		$Mo^{6+}(3d_{5/2})$
	235.9			$Mo^{6+}(3d_{3/2})$
K-MoO_x	230.9	76	5.24	$Mo^{5+}(3d_{5/2})$
	234.1			$Mo^{5+}(3d_{3/2})$
	232.3	24		$Mo^{6+}(3d_{5/2})$
	235.5			$Mo^{6+}(3d_{3/2})$
Al-MoO_x	230	13	5.1	$Mo^{4+}(3d_{5/2})$
	233.2			$Mo^{4+}(3d_{3/2})$
	231.05	64		$Mo^{5+}(3d_{5/2})$
	234.25			$Mo^{5+}(3d_{3/2})$
	232.3	23		$Mo^{6+}(3d_{5/2})$
	235.5			$Mo^{6+}(3d_{3/2})$

Table S2 The elemental contents in M-MoO_x measured by ICP

Electrode	Mo (mol kg^{-1})	Al (mol kg^{-1})	M (Li/Na/K) (mol kg^{-1})	Al/Mo molar ratio	M/Mo molar ratio
K-MoO_x	1.0213	-	0.4163	-	0.4076
Na-MoO_x	1.1011	-	0.4968	-	0.4512
Li-MoO_x	1.1443	-	0.4956	-	0.4331
Al-MoO_x	0.9815	0.1469	0.0108	0.1497	0.0110
Al-MoO_x-L	1.0534	0.0535	-	0.0508	-

Table S3 Mo 3d XPS results of the as-prepared Mg-MoO_x, Ca-MoO_x and Sr-MoO_x

Samples	Component (eV)	Content (%)	Average valence	Assignment
Mg-MoO_x	229.8<br>233	9	5.19	$Mo^{4+}(3d_{5/2})$<br>$Mo^{4+}(3d_{3/2})$
	231<br>234.2	63		$Mo^{5+}(3d_{5/2})$<br>$Mo^{5+}(3d_{3/2})$
	232.4<br>235.6	28		$Mo^{6+}(3d_{5/2})$<br>$Mo^{6+}(3d_{3/2})$
Ca-MoO_x	229.8<br>233	15	5.19	$Mo^{4+}(3d_{5/2})$<br>$Mo^{4+}(3d_{3/2})$
	231<br>234.2	51		$Mo^{5+}(3d_{5/2})$<br>$Mo^{5+}(3d_{3/2})$
	232.4<br>235.6	34		$Mo^{6+}(3d_{5/2})$<br>$Mo^{6+}(3d_{3/2})$
Sr-MoO_x	229.8<br>233	8	5.19	$Mo^{4+}(3d_{5/2})$<br>$Mo^{4+}(3d_{3/2})$
	231<br>234.2	65		$Mo^{5+}(3d_{5/2})$<br>$Mo^{5+}(3d_{3/2})$
	232.4<br>235.6	27		$Mo^{6+}(3d_{5/2})$<br>$Mo^{6+}(3d_{3/2})$

Table S4 Mo 3d XPS results of Al-MoO_x and MoO_x at different states of charge

Samples	Component (eV)	Content (%)	Average valence	Assignment
MoO_x-discharge	230.2<br>233.4	10	5.14	$Mo^{4+}(3d_{5/2})$<br>$Mo^{4+}(3d_{3/2})$
	231.3<br>234.5	66		$Mo^{5+}(3d_{5/2})$<br>$Mo^{5+}(3d_{3/2})$
	232.5<br>235.7	24		$Mo^{6+}(3d_{5/2})$<br>$Mo^{6+}(3d_{3/2})$
MoO_x-charge	230<br>233.2	8	5.29	$Mo^{4+}(3d_{5/2})$<br>$Mo^{4+}(3d_{3/2})$
	231.1<br>234.3	55		$Mo^{5+}(3d_{5/2})$<br>$Mo^{5+}(3d_{3/2})$
	232.3<br>235.5	37		$Mo^{6+}(3d_{5/2})$<br>$Mo^{6+}(3d_{3/2})$
Al-MoO_x-discharge	230.1<br>233.3	37	4.75	$Mo^{4+}(3d_{5/2})$<br>$Mo^{4+}(3d_{3/2})$
	231.15<br>234.35	51		$Mo^{5+}(3d_{5/2})$<br>$Mo^{5+}(3d_{3/2})$
	232.4<br>235.6	12		$Mo^{6+}(3d_{5/2})$<br>$Mo^{6+}(3d_{3/2})$
Al-MoO_x-charge	230.0<br>233.2	19	5.01	$Mo^{4+}(3d_{5/2})$<br>$Mo^{4+}(3d_{3/2})$
	231.05<br>234.25	61		$Mo^{5+}(3d_{5/2})$<br>$Mo^{5+}(3d_{3/2})$
	232.3<br>235.5	20		$Mo^{6+}(3d_{5/2})$<br>$Mo^{6+}(3d_{3/2})$

Table S5 The calculated total Gibbs free energy of protonated MoO_x and Al-MoO_x

Samples	MoO_x	Al-MoO_x
Total energy/ eV	-760.11	-792.06

Table S6 ICP results of Mo concentration in the electrolytes with the same volume after 7500 cycles

Samples	Electrolyte	Mo concentration/ mg L^{-1}
MoO_x	2 M $AlCl_3$	2.34
K-MoO_x	2 M KCl with the pH of 0.4	1.65
Al-MoO_x	2 M $AlCl_3$	1.41

Table S7 Mo 3d XPS results of the as-prepared samples after 7500 cycles

Samples	Component (eV)	Content (%)	Average valence	Assignment
K-MoO_x	230.15<br>233.35	13	5.11	$Mo^{4+}(3d_{5/2})$<br>$Mo^{4+}(3d_{3/2})$
	231.25<br>234.45	63		$Mo^{5+}(3d_{5/2})$<br>$Mo^{5+}(3d_{3/2})$
	232.5<br>235.7	24		$Mo^{6+}(3d_{5/2})$<br>$Mo^{6+}(3d_{3/2})$
Al-MoO_x	230.0<br>233.2	14	5.11	$Mo^{4+}(3d_{5/2})$<br>$Mo^{4+}(3d_{3/2})$
	231.1<br>234.3	66		$Mo^{5+}(3d_{5/2})$<br>$Mo^{5+}(3d_{3/2})$
	232.3<br>235.5	20		$Mo^{6+}(3d_{5/2})$<br>$Mo^{6+}(3d_{3/2})$

Table S8 The *i*H/*i*M ratios of M-MoO_x at 10th and 7500th cycles

Samples	10th cycle			7500th cycle		
	*i*H	*i*M	*i*H/*i*M	*i*H	*i*M	*i*H/*i*M
K-MoO_x	0.8952	2.0762	0.43	1.6141	0.7452	2.17
Al-MoO_x	0.9928	1.8385	0.54	0.9249	1.4745	0.63

Table S9 Performance comparison of different anode materials for half cells

Material	Electrolyte	Potential window (V)	Cycling number	Capacity fading per cycle (%)	Journal	Year
Al-MoO_x	2 M $AlCl_3$	0 ~ -0.7 (vs. SCE)	7500	0.0024		This work

PTCDA [S2]	1 M H_2SO_4	0 ~ -0.55 (vs. Ag/AgCl)	120	0.208	Angew. Chem. Int. Ed.	2017
MoO_3 [S3]	1 M H_2SO_4	0.3 ~ -0.5 (vs. Ag/AgCl)	100	0.33	Angew. Chem. Int. Ed.	2018
PTO [S4]	2 M H_2SO_4 + 2 M $MnSO_4$	0 ~ 0.7 (vs. Ag/AgCl)	1000	0.022	Nat. Commun.	2020
MoO_3 [S5]	9.5 M H_3PO_4	0.3 ~ -0.5 (vs. Ag/AgCl)	1000	0.018	Adv. Energy Mater.	2020
HATN [S6]	2 M $ZnSO_4$	0.3 ~ 1.1 (vs. Zn/Zn^{2+})	5000	0.0014	Angew. Chem. Int. Ed.	2020
MoO_3 [S7]	6 M H_2SO_4	0.3 ~ -0.5 (vs. SCE)	5000	0.0026	Adv. Funct. Mater.	2020
ALO [S8]	2 M HBF_4 + 2 M $Mn(BF_4)_2$	0.4 ~ -0.3 (vs. Ag/AgCl)	500	0.0228	Adv. Funct. Mater.	2021
MoO_3 [S9]	4.2 M H_2SO_4 + 5 M glucose	0.3 ~ -0.5 (vs. SCE)	500	0.026	Small	2021
MoO_3 [S10]	polyphosphoric acid	0.5 ~ -0.4 (vs. Ag/AgCl)	200	0.053	Nat. Commun.	2022
$H_{1.75}MoO_3$ [S11]	8 M H_2SO_4	0.7 ~ -0.38 (vs. Ag/AgCl)	5000	0.004	J. Am. Chem. Soc.	2022
MP [S12]	0.25 M $NH_4H_2PO_4$ + 0.25 M $(NH_4)_2HPO_4$	0 ~ -1 (vs. SCE)	2500	0.0048	Energy Storage Mater.	2022

Table S10 Performance comparison of different materials for PB full cells

Anode//Cathode	Voltage (V)	Energy Density (Wh/kg)	Cycling life/Capacity retention	Journal	Year
Al-MoO_x//MnO_2	1.37	160.2	1000/91.3		This work
PTO// MnO_2@GF [S4]	0.85	132.6	5000/80	Nat. Commun.	2020
MoO_3// MnO_2@GF [S13]	1.09	177.4	300/81	ACS Energy Lett.	2020

MoO_3//H-TBA [S5]	0.91	40	1000/85	Adv. Energy Mater.	2020
ALO//MnO_2/CF [S8]	1.2	174.6	300/68	Adv. Funct. Mater.	2021
PNAQ//PNAQ [S14]	0.65	55.4	500/70	Small Methods	2021
MoO_3//$LiVPO_4F$ [S10]	0.85	66.5	1000/∽100	Nat. Commun.	2022
$H_{1.75}MoO_3$//$H_{1.75}MoO_3$ [S11]	0.4	59	1000/75	J. Am. Chem. Soc.	2022
MP//Cu-HCF [S12]	1.3	55.3	500/75.7	Energy Storage Mater.	2022

Supplementary References

[S1] H.S. Kim, J.B. Cook, H. Lin, J.S. Ko, S.H. Tolbert et al., Oxygen vacancies enhance pseudocapacitive charge storage properties of MoO_{3-x}. Nat. Mater. **16**(4), 454-460 (2017). https://doi.org/10.1038/nmat4810

[S2] X. Wang, C. Bommier, Z. Jian, Z. Li, R. S. Chandrabose et al., Hydronium-ion batteries with perylenetetracarboxylic dianhydride crystals as an electrode. Angew. Chem. Int. Ed. **56**(11), 2909-2913 (2017). https://doi.org/10.1002/anie.201700148

[S3] X. Wang, Y. Xie, K. Tang, C. Wang, C. Yan, Redox chemistry of molybdenum trioxide for ultrafast hydrogen-ion storage. Angew. Chem. Int. Ed. **57**(36), 11569-11573 (2018). https://doi.org/10.1002/anie.201803664

[S4] Z. Guo, J. Huang, X. Dong, Y. Xia, L. Yan et al., An organic/inorganic electrode-based hydronium-ion battery. Nat. Commun. **11**(1), 959 (2020). https://doi.org/10.1038/s41467-020-14748-5

[S5] H. Jiang, W. Shin, L. Ma, J.J. Hong, Z. Wei et al., A high-rate aqueous proton battery delivering power below −78 °C via an unfrozen phosphoric acid. Adv. Energy Mater. **10**(28), 2000968 (2020). https://doi.org/10.1002/aenm.202000968

[S6] Z. Tie, L. Liu, S. Deng, D. Zhao, Z. Niu, Proton insertion chemistry of a zinc-organic battery. Angew. Chem. Int. Ed. **59**(12), 4920-4924 (2020). https://doi.org/10.1002/anie.201916529

[S7] Z. Su, W. Ren, H. Guo, X. Peng, X. Chen et al., Ultrahigh areal capacity hydrogen-ion batteries with MoO_3 loading over 90 mg cm^{-2}. Adv. Funct. Mater. **30**(46), 2005477 (2020). https://doi.org/10.1002/adfm.202005477

[S8] T. Sun, H. Du, S. Zheng, J. Shi, Z. Tao, High power and energy density aqueous proton battery operated at −90 °C. Adv. Funct. Mater. **31**(16), 2010127 (2021). https://doi.org/10.1002/adfm.202010127

[S9] Z. Su, J. Chen, W. Ren, H. Guo, C. Jia et al., "Water-in-sugar" electrolytes enable ultrafast and stable electrochemical naked proton storage. Small **17**(40), e2102375 (2021). https://doi.org/10.1002/smll.202102375

[S10] M. Liao, X. Ji, Y. Cao, J. Xu, X. Qiu et al., Solvent-free protic liquid enabling batteries operation at an ultra-wide temperature range. Nat. Commun. **13**, 6064 (2022). https://doi.org/10.1038/s41467-022-33612-2

[S11] W. Xu, K. Zhao, X. Liao, C. Sun, K. He et al., Proton Storage in Metallic $H_{1.75}MoO_3$ Nanobelts through the Grotthuss Mechanism. J. Am. Chem. Soc. **144**(38), 17407-

17415 (2022). https://doi.org/10.1021/jacs.2c03844

[S12] Z. Qin, Y. Song, Y. Liu, X. Liu, Accessing the proton storage in neutral buffer electrolytes using an electrodeposited molybdenum phosphate. Energy Storage Mater. **53**, 569-579 (2022). https://doi.org/10.1016/j.ensm.2022.09.035

[S13] L. Yan, J. Huang, Z. Guo, X. Dong, Z. Wang et al., Solid-state proton battery operated at ultralow temperature. ACS Energy Lett. **5**(2), 685-691 (2020). https://doi.org/10.1021/acsenergylett.0c00109

[S14] T. Sun, H. Du, S. Zheng, J. Shi, X. Yuan et al., Bipolar organic polymer for high performance symmetric aqueous proton battery. Small Methods **5**(8), e2100367 (2021). https://doi.org/10.1002/smtd.202100367
